# USP43 stabilizes c-Myc to promote glycolysis and metastasis in bladder cancer

**DOI:** 10.1038/s41419-024-06446-7

**Published:** 2024-01-13

**Authors:** Mingxing Li, Jingtian Yu, Lingao Ju, Yejinpeng Wang, Wan Jin, Renjie Zhang, Wan Xiang, Meng Ji, Wenzhi Du, Gang Wang, Kaiyu Qian, Yi Zhang, Yu Xiao, Xinghuan Wang

**Affiliations:** 1 Department of Urology, Laboratory of Precision Medicine, Zhongnan Hospital of Wuhan University, Wuhan, China; 2 Department of Biological Repositories, Human Genetic Resources Preservation Center of Hubei Province, Hubei Key Laboratory of Urological Diseases, Zhongnan Hospital of Wuhan University, Wuhan, China; 3 Euler Technology, ZGC Life Sciences Park, Beijing, China; 4 Department of Laboratory Medicine, Zhongnan Hospital of Wuhan University, Wuhan, China; 5 Department of Urology, The First Affiliated Hospital of Shandong First Medical University & Shandong Provincial Qianfoshan Hospital, Jinan, China; 6 Center for Quantitative Biology, School of Life Sciences, Peking University, Beijing, China; 7 Medical Research Institute, Frontier Science Center for Immunology and Metabolism, Wuhan University, Wuhan, China; 8 Wuhan Research Center for Infectious Diseases and Cancer, Chinese Academy of Medical Sciences, Wuhan, China

**Keywords:** Bladder cancer, Ubiquitylation, Oncogenes

## Abstract

A hallmark of tumor cells, including bladder cancer (BLCA) cells, is metabolic reprogramming toward aerobic glycolysis (Warburg effect). The classical oncogene MYC, which is crucial in regulating glycolysis, is amplified and activated in BLCA. However, direct targeting of the c-Myc oncoprotein, which regulates glycolytic metabolism, presents great challenges and necessitates the discovery of a more clarified regulatory mechanism to develop selective targeted therapy. In this study, a siRNA library targeting deubiquitinases identified a candidate enzyme named USP43, which may regulate glycolytic metabolism and c-Myc transcriptional activity. Further investigation using functional assays and molecular studies revealed a USP43/c-Myc positive feedback loop that contributes to the progression of BLCA. Moreover, USP43 stabilizes c-Myc by deubiquitinating c-Myc at K148 and K289 primarily through deubiquitinase activity. Additionally, upregulation of USP43 protein in BLCA increased the chance of interaction with c-Myc and interfered with FBXW7 access and degradation of c-Myc. These findings suggest that USP43 is a potential therapeutic target for indirectly targeting glycolytic metabolism and the c-Myc oncoprotein consequently enhancing the efficacy of bladder cancer treatment.

## Introduction

Globally, bladder cancer (BLCA) was estimated to account for 573,000 new cases and 213,000 deaths in 2020 [1]. Surgery in combination with adjuvant chemotherapy has improved the survival rates of patients with BLCA. However, the recurrence and aggressiveness of BLCA are major factors contributing to the poor prognosis [2]. Therefore, understanding the molecular regulatory network of BLCA is crucial for improving treatment options. Under normoxic conditions, cancer cells utilize glycolytic metabolism instead of oxidative phosphorylation to generate energy, which is referred to as aerobic glycolysis or the Warburg effect [3]. The Warburg effect is closely related to BLCA pathogenesis and aggressiveness [4].

c-Myc plays a vital role in regulating aerobic glycolysis. It directly activates the transcription of nearly all glycolytic genes by binding to the classical E-box sequence, including *GLUT1*, *HK2*, and *LDHA* [5]. Additionally, c-Myc can activate the transcription of splicing factors to enhance the expression of *PKM2* and promote glycolysis [6].

Proteins are the fundamental units of life activities, and ubiquitination is the second most common type of protein posttranslational modification. Deubiquitinases (DUBs) remove ubiquitin from ubiquitinated substrates, counteracting E3 ubiquitin ligase-mediated modification. The balance between the activities of E3 ubiquitin ligases and DUBs determines the fate of substrate proteins. Increasing evidence suggests that aberrant ubiquitination is critical in tumor development [7]. For instance, the E3 ubiquitin ligases NEDD4-1 and WWP2 catalyze PTEN polyubiquitination, leading to PTEN degradation and promoting tumor growth [8, 9]. Previously, our group discovered that the E3 ubiquitin ligase RNF126 affects BLCA progression by regulating PTEN stability [10]. In contrast to ubiquitin ligases, USP13 and OTUD3 stabilize PTEN by cleaving its polyubiquitin chains, inhibiting tumor progression [11, 12].

c-Myc is a highly active transcription factor in most human tumors that regulates a variety of tumor phenotypes, including proliferation, invasion, cell survival, genomic instability, angiogenesis, metabolism and immune evasion [13]. Amplification of the MYC oncogene has been observed in BLCA, and its products have been found to promote BLCA tumorigenesis [14–16]. Despite its importance in tumorigenesis, c-Myc is highly unstable and is rapidly degraded through the ubiquitin‒proteasome pathway [17]. Several E3 ubiquitin ligases, such as SKP2, FBXW7, CHIP, and FBXO32, have been shown to ubiquitinate and degrade c-Myc, thus suppressing tumorigenesis [18–21]. Conversely, USP37 and USP29 promote tumorigenesis by stabilizing c-Myc through deubiquitination [22, 23]. While c-Myc has been the focus of much research as a potential therapeutic target, its indirect targeting has gained attention due to its instability. One promising approach is to selectively inhibit deubiquitinases that stabilize c-Myc [24]. For example, P22077, a small molecule inhibitor of USP7, significantly inhibited neuroblastoma with MYCN amplification in a xenograft model [25]. Therefore, identifying more deubiquitinases of c-Myc can expand the possibilities for indirect inhibition of c-Myc to treat tumors. This study reveals that USP43 promotes aerobic glycolysis and metastasis in BLCA by stabilizing c-Myc, thus providing a novel target for indirect inhibition of c-Myc.

## Materials and methods

### Cell culture

T24 and 5637 cells were cultured in 1640 medium, UM-UC-3 cells were cultured in MEM, and 293 T cells were cultured in DMEM. The cells used in this study were obtained from the Chinese Academy of Sciences Cell Bank and were free of mycoplasma contamination. Cell lines were validated using the short tandem repeat (STR) method. All media were supplemented with 10% fetal bovine serum. All cells were cultured at 37 °C in an atmosphere of 5% CO_2_.

### siRNAs and plasmids

The human DUBs siRNA library (G-104705) was purchased from GE Healthcare Dharmacon. Small interfering RNAs (siRNAs) targeting *USP43*, *MYC* and *FBXW7* were synthesized by our commission from GenePharma (Suzhou, China). The siRNA sequences were as follows:

5’-GGUGGUCCUUUGGAUCCAATT-3’ (siUSP43-1);

5’-CCAGUUACCCGCUGGACUUTT-3’ (siUSP43-2);

5’-CGUUGUCUUGUAAUCUCUAAA-3’ (siUSP43^3’ UTR^);

5’-GCUUGUACCUGCAGGAUCUTT-3’ (siMYC-1);

5’-GGAAGAAAUCGAUGUUGUUTT-3’ (siMYC-2);

5’-GCAUAUGAUUUUAUGGUAATT-3’ (siFBXW7).

The Flag-USP43 plasmid was a gift from Professor Yongfeng Shang at Peking University. Flag-c-Myc and HA-c-Myc plasmids were gifts from Professor Guoliang Qing at Wuhan University. Molecular cloning was used for all other constructs, and DNA sequencing was performed to confirm their integrity.

### RNA extraction and quantitative reverse transcription PCR (qRT-PCR)

RNA was extracted and reverse transcribed, and a qRT-PCR protocol was carried out as described previously [26]. Supplementary Table S1 lists the qRT-PCR primer sequences used in this study.

### Western blot analyses

In general, cells were lysed with RIPA lysate containing a cocktail of protease inhibitors for 40 min on ice and then centrifuged at 14,000 g at 4 °C for 5 min. The supernatant was then removed, and 5× loading buffer was added and denatured at 100 °C for 10 min. Total proteins were separated by SDS-PAGE and then subjected to immunoblotting experiments with the corresponding antibodies as previously described [27]. Information about the primary antibodies used in the article is listed in Supplementary Table S2.

### Glucose consumption and lactate production measurement

In short, after 48 h of transfection, 1 million cells were collected and reseeded in 6-well plates. After 8 h of cell attachment, the cells were cultured in serum-free medium for another 24 h. Then, the supernatant was collected, and the glucose and lactate contents were measured by kits. A glucose assay kit (BioVision, #K606-100) was used to quantify glucose levels, and a lactate assay kit (BioVision, #K607-100) was used to determine lactate levels.

### Wound healing assay

The wound healing assay involved seeding cells in 6-well plates. After the cells became confluent, they were scratched out with a pipette tip, and the floating cells were washed off with PBS. Immediately after, the wound was photographed. Following 24 h of culture in serum-free medium, the wound was recorded again.

### Transwell migration assay

For the transwell migration assay, 40,000 cells mixed in 200 μL of serum-free medium were seeded in an upper chamber (Corning, USA). Then, 600 μL of complete medium was added to the bottom chambers. After 24 h of incubation, 30 min of fixation was followed by 1 h of staining with crystal violet for the migrated cells. After cleaning and drying, we photographed the cells under a microscope and counted them using ImageJ software.

### Construction of stable USP43 knockdown cell lines

Packaged lentiviruses were purchased from GenePharma (Suzhou, China) with the following sequences: 5’-TTCTCCGAACGTGTCACGT-3’ (shNC) and 5’-GGTGGTCCTTTGGATCCAA-3’ (shUSP43). Stable cell lines were constructed and selected according to the GenePharma Recombinant Lentivirus Operation Manual. The successful construction of stable cell lines was confirmed by qRT-PCR and Western blot analysis.

### Animal studies

We purchased 4-week-old male BALB/c nude mice from WQJX BioTechnology (Wuhan, China). After 1 week of adaptive feeding, the mice were randomly divided. To establish a nude mouse model of popliteal lymph node metastasis, 1 × 10^6^ stable T24 cells (T24-shNC or T24-shUSP43) were resuspended in 50 µL of sterile PBS and injected into the right footpads of nude mice. The nude mice were observed every 3 days and sacrificed after 4 weeks. The popliteal lymph nodes were dissected, and the volume was measured. Subsequent pathological and immunohistochemical analyses were then performed after fixation with paraformaldehyde. LN volume (mm^3^) = (length (mm)) × (width (mm))^2^ × 0.52. To establish a model of caudal vein-lung metastasis in nude mice, the nude mice were injected via the tail vein with 1 × 10^6^ stably transformed T24 cells resuspended in 100 μL PBS. We observed the nude mice every three days, and in vivo imaging of small animals was performed after 6 weeks to determine the status of lung metastasis. For further investigation, mice were sacrificed, and the lungs were dissected. Two groups of mice (*n* = 5 each) were maintained for survival analysis. The experiment ended when the subject died or survived 60 days. The investigator was blinded to the group allocation of the mice during the experiment. The sample size is described in the corresponding figure legend. No animals were excluded from the analysis.

### Coimmunoprecipitation assay

In brief, after lysis of the cells to obtain the supernatant, the indicated antibody was added for incubation overnight with shaking at 4 °C. The next morning, 20 µL of washed Protein A/G magnetic beads were added to the antigen-antibody complex system for continued incubation for 2 h. After washing three times with IP washing buffer, 1× loading buffer was added to the complex system and denatured at 100 °C for 5 min to separate the coprecipitated complexes. Then, the samples were subjected to immunoblot analysis.

### GST pull-down

The GST-tagged c-Myc protein and His-tagged USP43 protein were expressed and purified using the *E. coli* expression system. 2 μg of His-USP43 protein and 2 μg of GST or GST-c-Myc protein were mixed in IP binding buffer and incubated for 4 h with shaking at 4 °C. Subsequently, 30 μL of washed Glutathione Sepharose beads were added to continue the incubation for 2 h, and after three washes with IP washing buffer, the samples were resuspended in 1× loading buffer and denatured at 100 °C for 10 min for subsequent immunoblot analysis.

### Chromatin immunoprecipitation (ChIP)

The manufacturer’s instructions were followed when performing ChIP. In short, following fixation of 1 × 10^7^ cells with 1% formaldehyde for 10 min at 37 °C, 0.125 M glycine was added immediately to quench for 5 min at 37 °C before lysing in SDS lysis buffer. Afterwards, chromatin was fragmented by sonication and incubated overnight at 4 °C with anti-c-Myc antibody (Abcam, ab32072) or IgG (Proteintech, B900610) and Protein A/G magnetic beads. After washing the complexes with low-salt and high-salt solutions in turn, purified DNA was obtained for subsequent quantitative PCR (qPCR) analysis. The primer sequences for the USP43 promoter are shown in Supplementary Table S1.

### Dual-luciferase reporter assay

293 T cells were transfected with the indicated luciferase plasmids in 24-well plates, and luciferase activity was measured using the Dual-Luciferase^®^ Assay Kit (Promega, E1910). Firefly luciferase activities were normalized to Renilla luciferase control values.

### In vivo deubiquitination assay

After transfection as indicated, cells were treated with 10 μM MG132 for 6 h before harvesting and then lysed on ice for 40 min with RIPA lysate containing a cocktail of protease inhibitors. The supernatant was obtained after centrifugation at 4 °C for 10 min and incubated overnight with the corresponding antibody. The next morning, the system was added to washed Protein A/G magnetic beads for subsequent binding. Finally, polyubiquitinated c-Myc was analyzed by SDS-PAGE gel and immunoblotting.

### In vitro deubiquitination assay

HA-c-Myc and Myc-Ubiquitin plasmids were transfected into 293 T cells. 6 h before harvesting, 10 μM MG132 was added to treat cells. We lysed 293 T cells transfected with the GFP-USP43 plasmid and precipitated GFP-USP43 protein with an anti-GFP antibody. USP43 protein was obtained by nondenaturing elution. Polyubiquitinated c-Myc was incubated with or without the USP43 protein in deubiquitination buffer for 2 h at 37 °C. The buffer contained 50 mM Tris-HCl, 5 mM MgCl_2_, 2 mM DTT, and 2 mM ATP-Na_2_ with proteasome inhibitors.

### Statistical analysis

The statistical analysis was performed with GraphPad Prism version 9.0. Two-tailed Student’s t-test was used for comparisons between two groups. Statistical significance from three or more groups was calculated by one-way or two-way ANOVA with Tukey’s corrections. A log-rank test was used to estimate the significance of mouse survival. *p* < 0.05 was considered statistically significant.

## Results

### siRNA screening reveals USP43 as a key deubiquitinase for glycolysis and c-Myc transcriptional activity

**Fig. 1:**
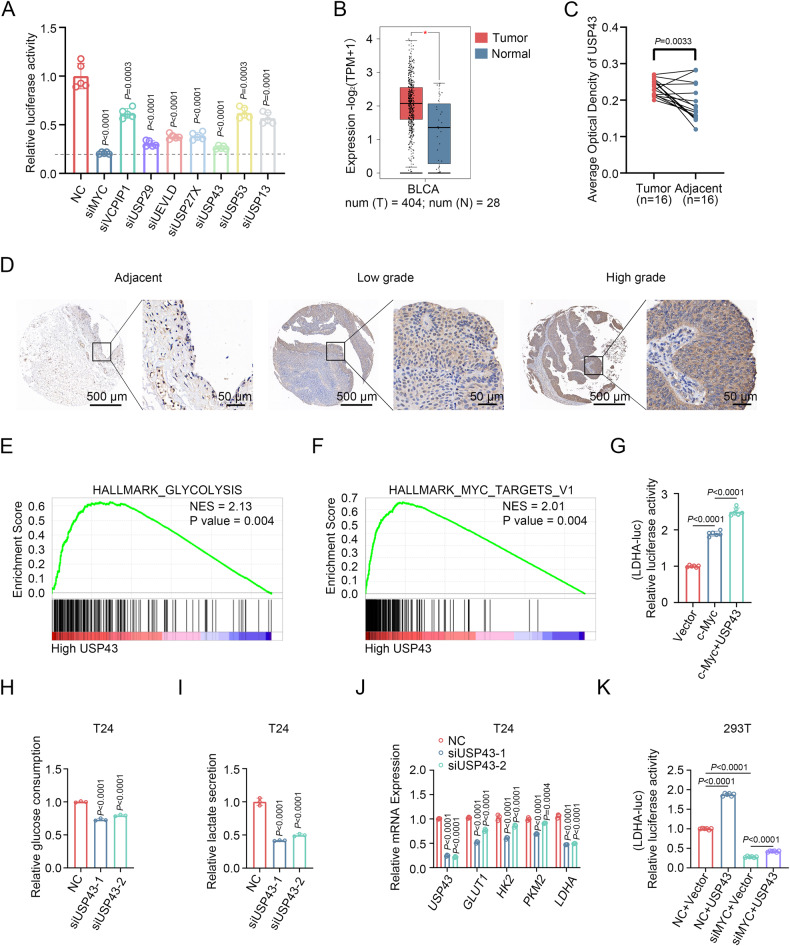
siRNA screening reveals USP43 as a key deubiquitinase for glycolysis and c-Myc transcriptional activity. **A** 293 T cells were transfected with siRNA pools targeting seven deubiquitinase candidates for 24 h, transfected with 5× E-box luciferase reporter for 36 h, and finally subjected to a dual-luciferase reporter assay. NC was the negative control, and *MYC* RNAi was the positive control (*n* = 5, one-way ANOVA followed by Tukey’s correction). **B** *USP43* expression levels in bladder cancer and normal tissue at GEPIA (http://gepia.cancer-pku.cn/index.html). |Log_2_FC| Cutoff: 0.5, *p*-value Cutoff: 0.01. **C** Immunohistochemical detection of USP43 in carcinoma samples paired with adjacent normal tissues in the BLCA tissue microarray. Average optical density values were calculated using ImageJ software (tumor *n* = 16, adjacent *n* = 16, paired two-tailed Student’s t-test). **D** Representative immunohistochemical image of BLCA tissue microarray showing USP43 content in normal adjacent tissues and low-grade and high-grade bladder cancer tissues. **E**, **F** GSEA enrichment of TCGA-BLCA samples shows that *USP43* is positively related to the glycolysis pathway (**E**) and MYC target pathway (**F**). **G** Dual-luciferase reporter assay in 293 T cells cotransfected with LDHA promoter plasmid and empty vector, c-Myc overexpression plasmid, or c-Myc overexpression plasmid plus USP43 overexpression plasmid (*n* = 6, one-way ANOVA followed by Tukey’s correction). **H**, **I** The media from T24 cells was collected for the analysis of glucose consumption (**H**) and lactate production (**I**) (**H**, **I**, *n* = 3, one-way ANOVA followed by Tukey’s correction). **J** T24 cells were transfected with two different siRNAs against *USP43* or with a control siRNA for 48 h. The mRNA levels of *USP43*, *GLUT*, *HK2*, *PKM2*, and *LDHA* were detected using qRT-PCR (*n* = 3, two-way ANOVA test followed by Tukey’s correction). **K** Dual-luciferase reporter assay in 293 T cells transfected as indicated, and LDHA promoter activity was measured (*n* = 6, one-way ANOVA test followed by Tukey’s correction). The *n* number represents n biologically independent experiments in each group. The data are presented as the mean ± SD (bar plots).

To identify deubiquitinases (DUBs) that play roles in both glycolysis and c-Myc transcriptional activity, we performed further screening of seven DUBs (Supplementary Fig. S1A) [23]. We constructed a plasmid with a 5× E-box sequence reflecting c-Myc transcriptional activity and found that knockdown of *USP43* showed the strongest inhibitory effect on the 5× E-box luciferase reporter, based on a luciferase reporter assay (Fig. 1A and Supplementary Fig. S1B, C). Only *USP43* was found to be upregulated in BLCA among the seven DUBs screened, and patients with high *USP43* expression had a worse prognosis than those with low expression (Fig. 1B, C and Supplementary Fig. S2A–H). Immunohistochemical analysis of the BLCA tissue microarray showed that the protein level of USP43 increased with increasing pathological grade (Fig. 1D and Supplementary Fig. S2I). GSEA enrichment analysis revealed that USP43 was positively correlated with the glycolysis pathway and MYC target pathway in the TCGA BLCA dataset (Fig. 1E, F). The dual-luciferase reporter assay showed that USP43 could further enhance the activation of LDHA-luciferase by c-Myc (Fig. 1G). We then measured the effect of USP43 on glycolysis in BLCA cells and found that both glucose consumption and lactate production were reduced after *USP43* knockdown in T24 and 5637 cells (Fig. 1H, I and Supplementary Fig. S3A, B). The mRNA levels of key glycolytic genes *GLUT1*, *HK2*, *PKM2*, and *LDHA* were also decreased after *USP43* knockdown (Fig. 1J and Supplementary Fig. S3C), consistent with the GEPIA website showing that *USP43* is positively correlated with *GLUT1*, *HK2*, *PKM2*, and *LDHA* at the mRNA level (Supplementary Fig. S3D–G). The protein levels of GLUT1 and LDHA also showed the same trend (Supplementary Fig. S3H, I). Knockdown of *MYC* attenuated the enhancement of LDHA promoter activity by USP43 (Fig. 1K). Moreover, in the absence of *MYC*, the mRNA levels of four glycolytic enzymes were not affected by *USP43* depletion (Supplementary Fig. S3J). These results demonstrate that USP43 positively regulates glycolysis in BLCA and c-Myc transcriptional activity.

### USP43 regulates the metastasis of BLCA cells

**Fig. 2:**
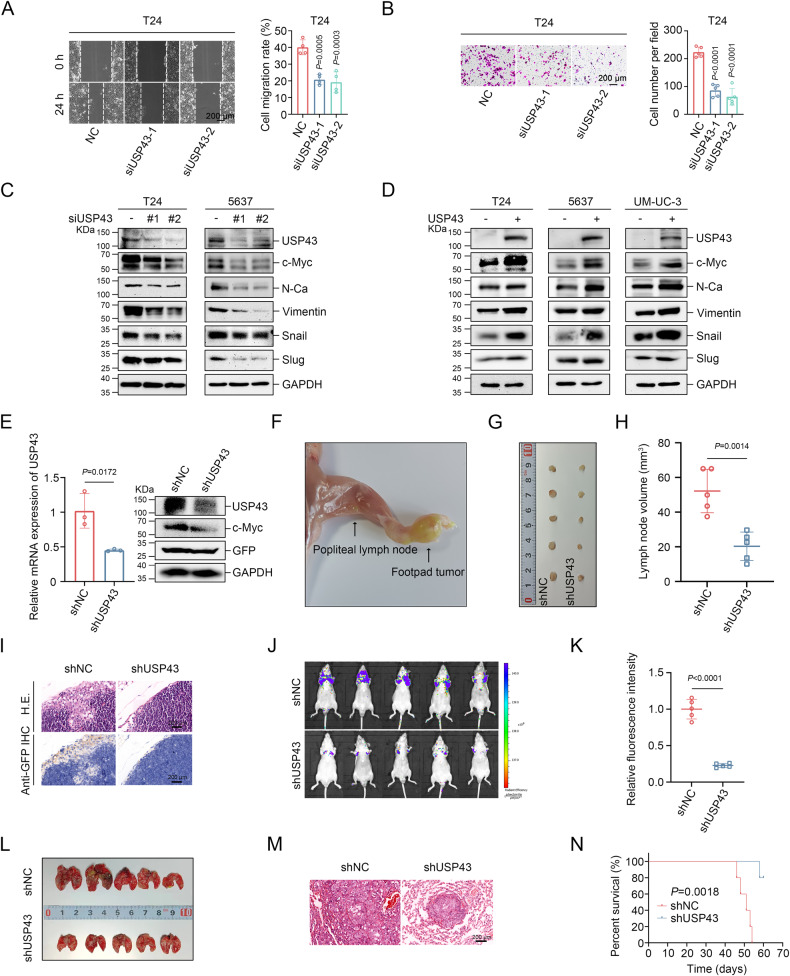
Knockdown of *USP43* suppresses BLCA cell metastasis. **A** Wound healing assay demonstrated that knockdown of *USP43* inhibited the migration ability of T24 cells (*n* = 4, one-way ANOVA test followed by Tukey’s correction). Migration rate = (wound area (0 h) – wound area (24 h))/wound area (0 h). **B** Transwell migration assays demonstrated that knockdown of *USP43* inhibited the migration ability of T24 cells (*n* = 5, one-way ANOVA followed by Tukey’s correction). The scale bar is 200 μm. **C**, **D** c-Myc and epithelial-mesenchymal transition-related proteins were detected by immunoblot assays following *USP43* knockdown (**C**) and overexpression (**D**). **E** The knockdown efficiency of *USP4*3 in T24 stable cell lines was verified by qRT-PCR (left) (*n* = 3, unpaired two-tailed Student’s t-test) and Western blot analysis (right). **F** Representative image of the popliteal lymph node metastasis model. **G**, **H** Images of dissected popliteal lymph nodes (**G**) and lymph node volumes (H) in each group (**H**, *n* = 5 for BALB/c nude mice, unpaired two-tailed Student’s t-test). **I** Representative images of the popliteal lymph nodes analyzed by H&E staining and IHC staining using an anti-GFP antibody. The scale bar is 200 μm. **J**, **K** Images of lung fluorescence after T24-shNC/T24-shUSP43 cells were injected into the tail veins of NOD/SCID mice for six weeks (**J**) and quantitation of the fluorescence intensity of lung metastases (**K**) (**J**, **K**, *n* = 5 for BALB/c nude mice, unpaired two-tailed Student’s t-test). **L**, **M** Images of dissected whole lungs (**L**) and representative H&E-stained lung tissue sections (**M**). The scale bar is 200 μm. **N** Kaplan-Meier survival curves were generated for NOD/SCID mice within 60 days after tail vein injection of T24-shNC/T24-shUSP43 cells (*n* = 5 for BALB/c nude mice, log-rank test). The *n* number represents n biologically independent experiments in each group. The data are presented as the mean ± SD (bar plots).

Next, the effect of USP43 on BLCA cells migration was examined. Specifically, we evaluated USP43’s effect on T24, 5637, and UM-UC-3 migration ability using wound healing and transwell migration assays. Our findings showed that the knockdown of *USP43* significantly inhibited the migration ability of these cell lines (Fig. 2A, B and Supplementary Fig. S4A–D), while overexpression of USP43 promoted migration (Supplementary Fig. S4E–H). Additionally, we explored the potential involvement of the epithelial-mesenchymal transition (EMT) pathway in tumor metastasis by assessing EMT-related markers using immunoblotting. The N-cadherin, Vimentin, Snail, and Slug protein levels were downregulated upon *USP43* knockdown and upregulated upon USP43 overexpression (Fig. 2C, D).

To further investigate the impact of USP43 on BLCA metastasis in vivo, we established stable *USP43* knockdown cell lines using lentiviral transfection methods and confirmed successful knockdown through qRT-PCR and immunoblot analysis (Fig. 2E). We then established popliteal lymph node metastasis and lung metastasis nude mouse models. Compared with those in mice inoculated with control T24 stable cell lines (LV-shNC cells), popliteal lymph node volume and metastatic lesions in popliteal lymph nodes were lower in nude mice inoculated with T24 stable *USP43* knockdown cell lines (LV-shUSP43 cells) (Fig. 2F–I). Additionally, in vivo imaging of the lung metastasis nude mouse model revealed that the fluorescence intensity in the lungs was downregulated in nude mice inoculated with LV-shUSP43 cells. This was consistent with the presentations of dissected lung tissues and pathological sections (Fig. 2J–M). Notably, the prognosis of nude mice inoculated with LV-shUSP43 cells was better than that of mice inoculated with LV-shNC cells (Fig. 2N). Overall, these findings suggest that USP43 promotes BLCA cell metastasis.

### USP43 stabilizes c-Myc

**Fig. 3:**
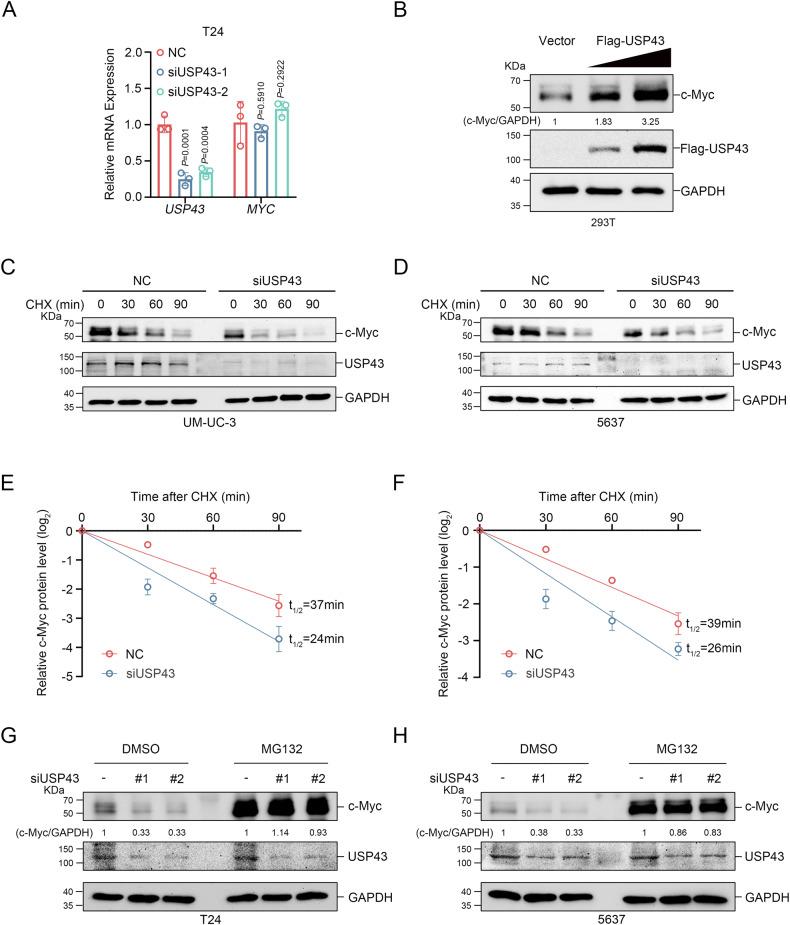
USP43 regulates c-Myc stability. **A** After knocking down *USP43* in T24 cells, the mRNA level was detected by qRT-PCR (*n* = 3, two-way ANOVA followed by Tukey’s correction). **B** 293 T cells were transfected with empty vector or with increasing concentrations of USP43 and c-Myc was detected by subsequent immunoblot analysis. **C**–**F** 48 h after *USP43* knockdown, UM-UC-3 (**C**) and 5637 (**D**) cells were treated with 50 μg/mL CHX and then harvested at the indicated time points. The statistical plot (**E**, **F**) represents the intensity of c-Myc bands detected by Western blot analysis (**C**, **D**, *n* = 3). **G**, **H** After 48 h of transfection, T24 (**G**) and 5637 (**H**) cells were treated with 10 μM MG132 for 6 h before harvest, and then c-Myc was detected by Western blot analysis. The *n* number represents n biologically independent experiments in each group. The data are presented as the mean ± SD (bar plots).

We next investigated whether USP43 is a DUB of c-Myc. Our results demonstrated that *USP43* knockdown and overexpression had no effect on *MYC* mRNA levels in T24 cells (Fig. 3A and Supplementary Fig. S5A). Similar findings were obtained in 5637 cells (Supplementary Fig. S5B). However, knockdown of *USP43* decreased the c-Myc protein level in T24 and 5637 cells (Fig. 2C), while overexpression of USP43 increased the c-Myc protein level in T24, 5637, and UM-UC-3 cells (Fig. 2D). Additionally, c-Myc protein levels were increased by USP43 in a dose-dependent manner (Fig. 3B). To determine whether USP43 affects the protein stability of c-Myc, a cycloheximide (CHX) chase assay was conducted in UM-UC-3 and 5637 cells transfected with *USP43* siRNA or USP43 overexpression plasmids. As expected, the half-life of c-Myc was significantly downregulated upon *USP43* knockdown and significantly upregulated upon USP43 overexpression (Fig. 3C–F and Supplementary Fig. S5C–F). Furthermore, the effect of USP43 on c-Myc was blocked by the proteasome inhibitor MG132 (Fig. 3G, H). Together, these results suggest that USP43 stabilizes c-Myc through the ubiquitin-proteasome pathway.

### USP43 interacts with c-Myc

**Fig. 4:**
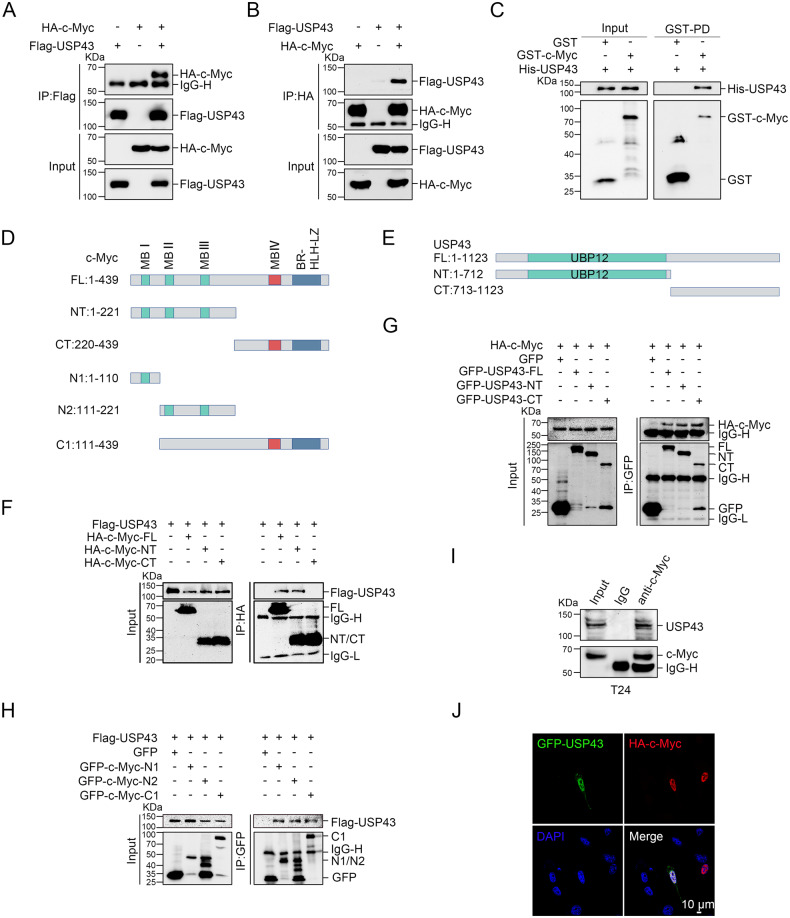
USP43 interacts with c-Myc. **A**, **B** Co-IP assay showed that exogenous USP43 interacted with c-Myc in 293 T cells. USP43 and c-Myc were precipitated with the corresponding anti-Flag (**A**) and anti-HA antibodies (**B**), respectively. **C** Recombinant purified GST-tagged c-Myc protein and His-tagged USP43 protein from *E. coli* were subjected to a GST pull-down assay in vitro. **D**, **E** Schematic diagram of c-Myc (**D**) and USP43 (**E**) truncations. **F**, **H** Flag-USP43 was cotransfected with c-Myc truncation mutants. Interactions were analyzed using the Co-IP assay. **G** HA-c-Myc was cotransfected with USP43 truncation mutants. Interactions were analyzed using the Co-IP assay. **I** c-Myc in T24 cell lysates was precipitated by c-Myc antibody. The interaction between endogenous USP43 and c-Myc was examined by Western blot analysis. **J** USP43 and c-Myc were transfected into UM-UC-3 cells for immunofluorescence assays. Colocalization analysis showed that USP43 and c-Myc colocalized in the nucleus. The scale bar is 10 μm.

Next, we performed a coimmunoprecipitation assay to investigate the potential interaction between USP43 and c-Myc. Our results demonstrated a strong interaction between exogenously expressed Flag-USP43 and HA-c-Myc in 293 T cells (Fig. 4A, B), while recombinant GST-c-Myc pulled down recombinant His-USP43 in vitro (Fig. 4C). To further explore the interacting domains of USP43 and c-Myc, we generated a series of truncated mutants and performed a coimmunoprecipitation assay. As shown in Fig. 4D–H, USP43 interacted with the N-, N1-, N2-, and C1-terminus of c-Myc, while c-Myc interacted with both the N-terminus and the C-terminus of USP43. Moreover, endogenous USP43 interacted with c-Myc in T24 and 5637 cells (Fig. 4I and Supplementary Fig. S6A). Immunofluorescence analysis revealed that c-Myc and USP43 were colocalized in the nucleus of UM-UC-3 cells (Fig. 4J). These findings indicate the interaction between USP43 and c-Myc.

### USP43 deubiquitinates c-Myc at K148 and K289

**Fig. 5:**
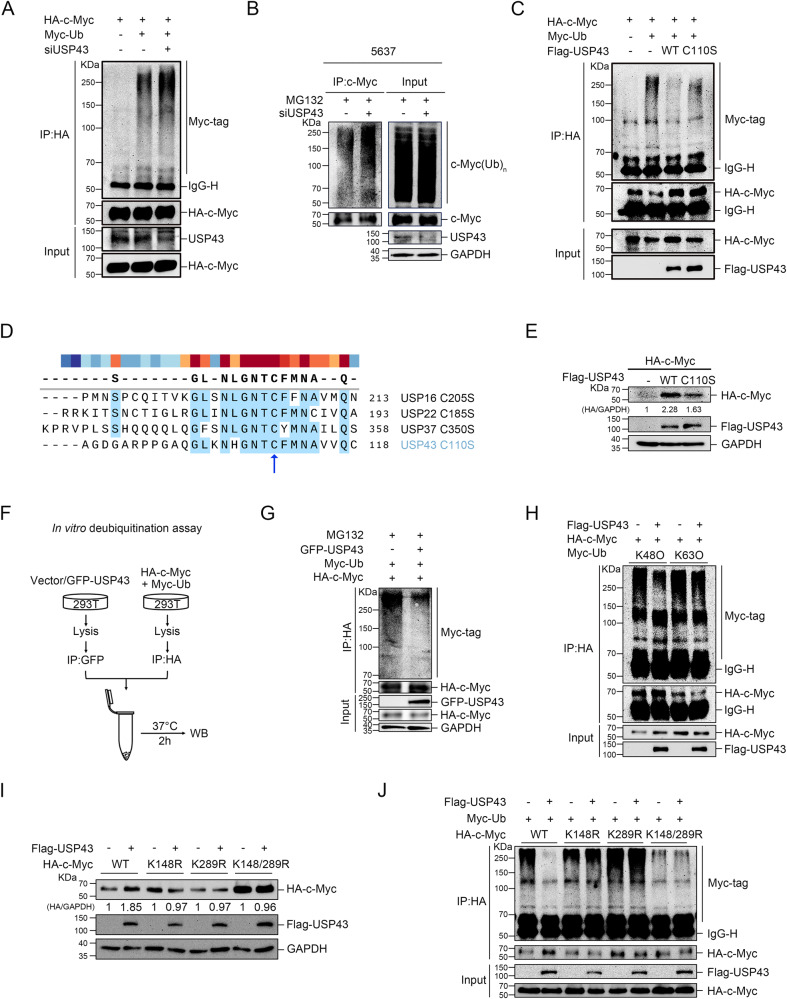
USP43 deubiquitinates c-Myc at K148 and K289. **A** 293 T cells were transfected with the indicated siRNAs and plasmids. Cells were treated with 10 μM MG132 for 6 h before harvest, and then ubiquitination experiments were performed to analyze polyubiquitination of c-Myc. **B** 5637 cells transfected with the indicated siRNA were treated with 10 μM MG132 for 6 h before collection. c-Myc was immunoprecipitated with anti-c-Myc and immunoblotted with anti-ubiquitin. **C** 293 T cells were transfected with HA-c-Myc, Myc-Ubiquitin and Flag-USP43 (wild-type or C110S). Cells were treated with 10 μM MG132 for 6 h before harvest, and then ubiquitination experiments were performed to analyze polyubiquitination of c-Myc. **D** Sequence alignment was used to determine the enzyme inactivating mutation site of USP43. **E** HA-c-Myc was cotransfected with wild-type USP43 or an enzyme inactivating mutant USP43 (C110S) into 293 T cells and subsequent immunoblot analysis. **F** Schematic diagram of the in vitro ubiquitination experimental procedure. **G** Deubiquitination of c-Myc in vitro by GFP-USP43. Polyubiquitinated c-Myc was lysed from 293 T cells transfected with HA-c-Myc and Myc-Ubiquitin plasmids and precipitated with anti-HA antibodies. 293 T cells transfected with GFP-USP43 plasmid were lysed and precipitated with anti-GFP antibodies followed by nondenaturing elution to obtain GFP-USP43. Polyubiquitinated c-Myc was incubated with or without GFP-USP43 and then analyzed using IB with anti-Myc antibodies. **H** 293 T cells were transfected with HA-c-Myc, Myc-Ubiquitin (K48O or K63O) and Flag-USP43. Cells were treated with 10 μM MG132 for 6 h before harvest, and then ubiquitination experiments were performed to analyze polyubiquitination of c-Myc. **I** HA-tagged wild-type c-Myc and three HA-tagged c-Myc mutants were cotransfected with Flag-USP43 into 293 T cells, and the expression of wild-type c-Myc and c-Myc mutants was detected by an anti-HA antibody. **J** The effect of USP43 on the ubiquitination level of wild-type c-Myc and three c-Myc mutants was analyzed by deubiquitination assay.

We next examined the impact of USP43 on c-Myc polyubiquitination levels. Our results demonstrated that c-Myc polyubiquitination was upregulated upon *USP43* knockdown and downregulated upon USP43 overexpression (Fig. 5A–C). To determine whether USP43 deubiquitinase activity is necessary for c-Myc regulation, we generated a USP43 deubiquitinase inactivating mutant by mutating the cysteine at position 110 to serine (C110S) (Fig. 5D). We found that while the C110S mutant had a similar c-Myc binding capacity to wild-type USP43, it was less effective at reducing c-Myc polyubiquitination levels and increasing c-Myc protein content (Fig. 5C, E, and Supplementary Fig. S6B). A similar trend was also observed in the activity of c-Myc on LDHA-luciferase (Fig. S6C). In vitro deubiquitination experiments confirmed these findings (Fig. 5F, G). Next, the type of polyubiquitin chain of c-Myc deubiquitinated by USP43 was examined, and our data indicated that USP43 catalyzed the deubiquitination of the K48 chain but not the K63 chain of c-Myc, which is associated with proteasomal pathway degradation [28] (Fig. 5H). To identify the lysine residue of c-Myc catalyzed by USP43, we constructed a series of c-Myc point mutant plasmids with lysine mutated to arginine. In the first round of screening, we found four c-Myc mutants (K148R, K289R, K371R, K397R) that were not regulated by USP43 (Supplementary Fig. S6D). In the second round of screening, we found only two c-Myc mutants (K148R and K289R) that were not regulated by USP43 (Supplementary Fig. S6E). We then constructed a double mutant of c-Myc and confirmed that K148 and K289 are indeed essential for USP43 catalysis of c-Myc (Fig. 5I and Supplementary Fig. S6F). Further deubiquitination assays showed that USP43 was able to deubiquitinate wild-type c-Myc but not the c-Myc mutants K148R, K289R, and K148/289R (Fig. 5J). Coimmunoprecipitation assays indicated that several c-Myc mutants could interact with USP43, similar to wild-type c-Myc (Supplementary Fig. S6G). These results suggest that USP43 deubiquitinates c-Myc at K148 and K289.

### USP43 antagonizes c-Myc degradation by FBXW7

**Fig. 6:**
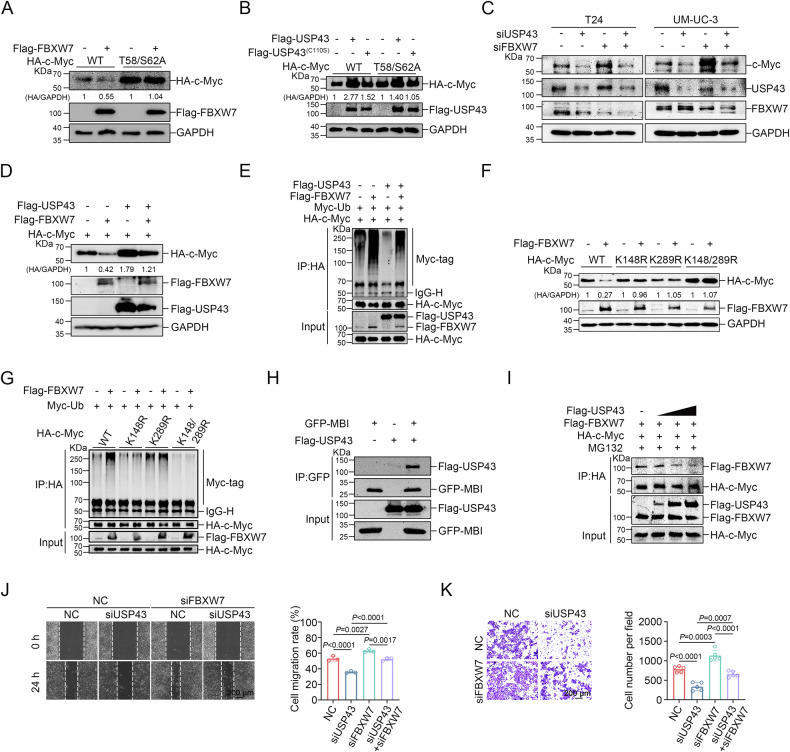
USP43 antagonizes c-Myc degradation by FBXW7. **A** HA-tagged wild-type or mutant c-Myc was cotransfected with Flag-tagged FBXW7 into 293 T cells, and HA-c-Myc was detected using Western blot analysis. **B** HA-tagged wild-type or mutant c-Myc was cotransfected with Flag-tagged wild-type or mutant USP43 into 293 T cells, and HA-c-Myc was detected using Western blot. **C** T24 and 5637 cells were transfected with siRNA against *USP43* or *FBXW7* as indicated. The protein levels were detected by Western blot analysis. **D** 293 T cells cotransfected with HA-c-Myc and Flag-FBXW7 or Flag-USP43 were lysed, and then the protein level was detected by Western blot analysis. **E** 293 T cells were transfected as indicated and treated with 10 μM MG132 for 6 h before harvest. The effect of FBXW7 or USP43 on the ubiquitination level of c-Myc was analyzed by ubiquitination assay. **F** The effect of FBXW7 on HA-tagged wild-type and three mutant c-Myc proteins was examined by Western blot. **G** The effect of FBXW7 on the ubiquitination level of wild-type c-Myc and three c-Myc mutants was analyzed by ubiquitination assay. **H** A co-IP assay was used to detect the interaction between the MBI domain of c-Myc and USP43. **I** Co-IP assay to detect the interaction between c-Myc and FBXW7 in the presence of USP43. 293 T cells were transfected with plasmids expressing c-Myc and FBXW7 and increasing amounts of USP43. **J**, **K** 5637 cells were treated with siRNA targeting *USP43* or *FBXW7* as indicated. Wound healing (**J**) (*n* = 3) and transwell migration assays (**K**) (*n* = 5) were performed to detect changes in cell migration ability (**J**, **K**, one-way ANOVA followed by Tukey’s correction). The scale bar is 200 μm. Migration rate = (wound area (0 h) – wound area (24 h)) / wound area (0 h). The *n* number represents n biologically independent experiments in each group. The data are presented as the mean ± SD (bar plots).

Previous reports have demonstrated that USP28 and USP38 stabilize c-Myc through the E3 ubiquitin ligase FBXW7 [29, 30]. Therefore, we examined whether USP43 could stabilize c-Myc by a similar mechanism. Sequential phosphorylation at serine 62 and serine 58 of c-Myc is required for FBXW7-mediated degradation of c-Myc. When serine 62 or serine 58 was mutated to alanine, FBXW7 failed to degrade c-Myc [19, 31]. As reported, FBXW7 was not capable of degrading c-Myc T58A, S62A, or T58/S62A mutants in comparison with wild-type c-Myc (Fig. 6A and Supplementary Fig. S7A). However, USP43 was able to stabilize not only wild-type c-Myc but also all these mutants (Fig. 6B and Supplementary Fig. S7B). Knockdown or overexpression of USP43 did not change the protein level of FBXW7 in T24 and 5637 cell lines (Supplementary Fig. S7C, D). Furthermore, knockdown of *USP43* in *FBXW7*-depleted T24 and 5637 cells still resulted in a reduction in c-Myc protein (Fig. 6C). Based on these results, it appears that USP43 stabilizes c-Myc independently of FBXW7.

We also investigated whether USP43 and FBXW7 could balance the protein levels of c-Myc. Our results showed that exogenous USP43 blocked the ubiquitination-mediated degradation of c-Myc by FBXW7 (Fig. 6D, E). USP43 deubiquitinates c-Myc at K148 and K289, and the K148/K289R c-Myc mutant showed an upregulated protein level and a downregulated ubiquitination level compared with wild-type c-Myc (Fig. 5I, J and Fig. 6F, G). Considering these findings, it was hypothesized that FBXW7 may catalyze c-Myc at K148 and K289. Indeed, FBXW7 was almost completely incapable of degrading c-Myc by ubiquitination when lysine 148 and 289 were mutated to arginine (K148R, K289R, K148/289R) but still retained the ability to bind to several c-Myc mutants (Fig. 6F, G and Supplementary Fig. S7E, F). Interestingly, wild-type USP43 can still stabilize T58A, S62A, and T58/S62A c-Myc mutants, which cannot be degraded by FBXW7 (Fig. 6B and Supplementary Fig. S7B), suggesting the existence of other E3 ubiquitin ligases that can catalyze c-Myc ubiquitination at K148 and K289.

Next, we examined the effect of SKP2, another E3 ubiquitin ligase of c-Myc, on K148R, K289R, and K148/289R c-Myc mutants. We found that these mutants impaired the SKP2-mediated degradation of c-Myc when compared with wild-type c-Myc, indicating that K148 and K289 are the less dominant sites for SKP2 to catalyze c-Myc ubiquitination (Supplementary Fig. S7F).

In our investigation of the impact of the DUB dead mutant USP43 (C110S) on wild-type c-Myc and T58A, S62A, and T58/S62A c-Myc mutants, we made an intriguing discovery. Specifically, we found that the C110S mutant was able to increase the wild-type c-Myc protein level, but not the T58A, S62A, and T58/S62A c-Myc mutants (Fig. 6B and Supplementary Fig. S7G). This suggests that when USP43 loses its deubiquitinase activity, it may rely on FBXW7 to regulate c-Myc. Since both USP43 and FBXW7 can bind to c-Myc, we hypothesized that they might compete for binding to c-Myc.

There has been evidence that FBXW7 binds to the Myc box I (MBI) of c-Myc in previous studies [31], and we wondered whether USP43 also had the ability to bind to the MBI of c-Myc. Our coimmunoprecipitation assay demonstrated that the MBI domain of c-Myc can indeed interact with USP43, and the interaction was weakened when the MBI domain was deficient in the c-Myc mutant (Fig. 6H and Supplementary Fig. S7H). Thus, the MBI domain appears to be a key interaction domain for c-Myc and USP43. Given that both USP43 and FBXW7 bind to the MBI of c-Myc, it seems reasonable to assume that increased USP43 may impede FBXW7’s access to c-Myc. In support of this notion, we observed that increasing doses of USP43 decreased FBXW7 binding to c-Myc (Fig. 6I). These results suggest that wild-type USP43 primarily stabilizes c-Myc by directly deubiquitinating c-Myc, while USP43 with a loss of catalytic activity still protects c-Myc to some extent by competitively binding to c-Myc with FBXW7. Functionally, USP43 and FBXW7 counteracted each other to influence the migration ability of 5637 cells (Fig. 6J, K). Overall, our results indicate that USP43 antagonizes the FBXW7-mediated degradation of c-Myc and cooperates with FBXW7 to regulate the migration ability of BLCA cells.

### USP43 is a direct target of c-Myc

**Fig. 7:**
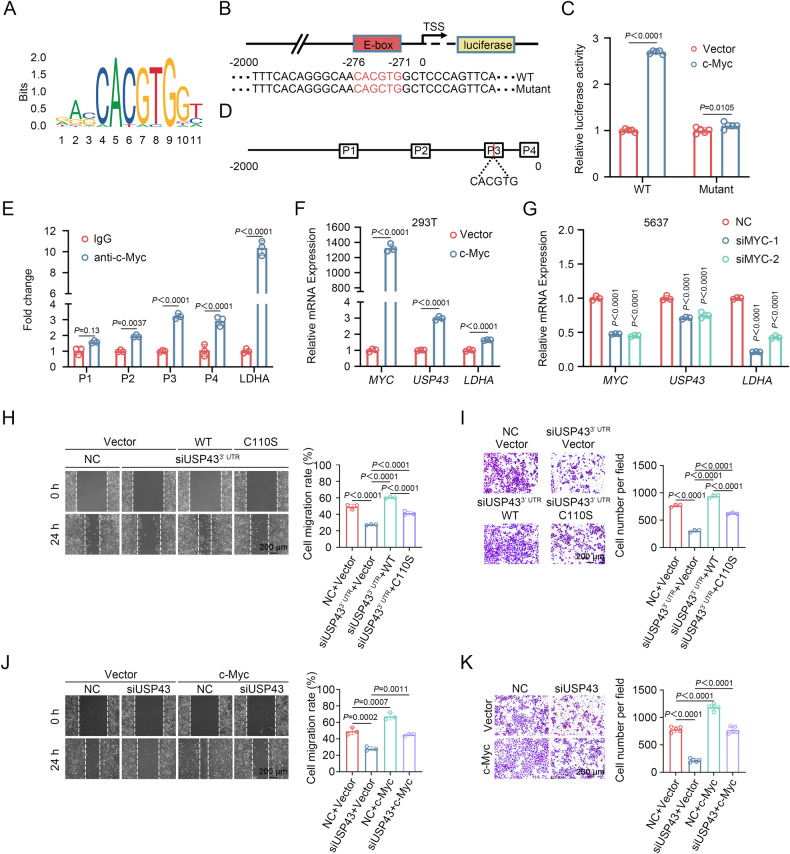
USP43 is a direct target of c-Myc. **A** The binding sites of c-Myc on promoter sequences obtained from the JASPAR database. **B** Schematic diagram of the potential sequence of c-Myc at the USP43 promoter binding site and the constructed luciferase plasmids containing wild-type or mutant USP43 promoter sequences. **C** Dual-luciferase reporter assay in 293 T cells cotransfected with USP43 promoter plasmid (wild-type or mutant) and empty vector or c-Myc overexpression plasmid (*n* = 5, two-way ANOVA followed by Tukey’s correction). **D** Schematic diagram of primers designed for ChIP-qPCR on the USP43 promoter sequence. **E** ChIP-qPCR analysis showed the enrichment degree of c-Myc in different regions of the USP43 promoter. IgG indicates the negative control (*n* = 3, two-way ANOVA followed by Tukey’s correction). **F** The c-Myc plasmid was transfected into 293 T cells, and then the mRNA levels were detected by qRT-PCR (*n* = 3, unpaired two-tailed Student’s t-test). **G** 5637 cells were transfected with siRNA targeting *MYC*, and mRNA levels were detected by qRT-PCR (*n* = 3, two-way ANOVA test followed by Tukey’s correction). **H**–**I** 5637 cells were treated with siRNA targeting *USP43* 3’ UTR followed by reconstitution with wild-type USP43 and a USP43 deubiquitinase inactivating mutant as indicated. Wound healing assays (**H**) (*n* = 3) and Transwell migration assays (**I**) (*n* = 3) were performed to detect changes in cell migration ability (**H**, **I**, one-way ANOVA followed by Tukey’s correction). The scale bar is 200 μm. **J**, **K** 5637 cells were treated with siRNA targeting *USP43* or c-Myc overexpression plasmid as indicated. Wound healing assays (**J**) (*n* = 3) and Transwell migration assays (**K**) (*n* = 5) were performed to detect changes in cell migration ability (**H**, **I**, one-way ANOVA followed by Tukey’s correction). The scale bar is 200 μm. The *n* number represents n biologically independent experiments in each group. The data are presented as the mean ± SD (bar plots).

c-Myc forms a heterodimer with MAX to activate the transcription of numerous genes by binding to the E-box sequence (CACGTG) of target genes, participating in various physiological and pathological processes [32]. The JASPAR database indicates a potential E-box sequence in the USP43 promoter region (Fig. 7A). Furthermore, ChIP-seq data from the GTRD and hTFtarget databases suggest that USP43 is a potential target gene of c-Myc (Supplementary Fig. S8A). The Cistrome Data Browser database also revealed a binding peak of c-Myc in the promoter region of USP43 (Supplementary Fig. S8B). Therefore, we aimed to investigate whether USP43 is transcriptionally regulated by c-Myc. First, the USP43 promoter, which spans -2000 to -1 (the translation initial site is 0), was amplified and then cloned into the pGL4.10 vector. The dual-luciferase reporter assay revealed that exogenous c-Myc significantly increased the activity of the USP43 promoter. However, when we mutated the E-box of the USP43 promoter to CAGCTG, the effect of exogenous c-Myc on mutant USP43 promoter activity was greatly weakened (Fig. 7B, C). To test whether c-Myc directly binds to the USP43 promoter, we performed chromatin immunoprecipitation (ChIP) and qPCR. The results showed that c-Myc could pull down the DNA fragment of the USP43 promoter region (Fig. 7D, E). Moreover, in 293 T cells, exogenous expression of c-Myc increased the mRNA level of *USP43* (Fig. 7F). Consistently, knockdown of *MYC* resulted in downregulation of *USP43* mRNA levels in 5637 and UM-UC-3 cells, and the protein levels of USP43 in 5637 cells showed the same trend (Fig. 7G and Supplementary Fig. S8C, D). Collectively, these data suggest that c-Myc can directly bind to the promoter region of USP43 and activate USP43 transcription, confirming that c-Myc is a transcription factor for USP43.

### USP43 regulates BLCA cell migration by targeting c-Myc

Because USP43 regulates c-Myc stability, we investigated whether the impact of USP43 on BLCA cell migration depended on c-Myc. Transwell migration and wound healing assays were conducted to assess the effects. We first knocked down *USP43* in 5637 cells, followed by reconstitution with wild-type USP43 or a deubiquitinase inactivating mutant. The results showed that the introduction of wild-type USP43 significantly reversed the inhibition of migration caused by *USP43* knockdown, while the deubiquitinase inactivating mutant only partially rescued the migration inhibition (Fig. 7H, I and Supplementary Fig. S9A). Next, we explored whether c-Myc could restore the migration inhibition caused by *USP43* knockdown. Our findings indicated that overexpression of c-Myc counteracted the reduced migration ability of 5637 and T24 cells resulting from *USP43* knockdown (Fig. 7J, K and Supplementary Fig. S9B, C). Therefore, we concluded that USP43 regulates BLCA cell migration primarily through c-Myc.

## Discussion

The initiation and progression of cancer are characterized by distinct metabolic reprogramming, which maintains the high proliferation rate of cancer cells [33]. In BLCA, various metabolic pathways are altered and contribute to tumorigenesis. Notably, a shift toward aerobic glycolytic metabolism (known as the Warburg effect) is a hallmark of tumor cells, including those found in BLCA. In response to high lactate content and subsequent acidification due to a glycolytic metabolic shift, carcinogenesis is facilitated by invasiveness, acid-mediated matrix degradation and metastasis [4]. Thus, the Warburg effect is associated with BLCA progression and aggressiveness. Oncogenes and tumor suppressors directly mediate the metabolic reprogramming of cancer cells. c-Myc, L-Myc, and N-Myc, which are members of the MYC family of oncoproteins, regulate metabolic reprogramming in human cancers. Although c-Myc expression is tightly regulated in normal cells, deregulation of c-Myc occurs in up to 70% of human cancers due to multiple mechanisms, including the gain in genetic copy number (chromosomal amplification or translocation), activation of superenhancers, aberrant upstream signaling, and altered protein stability [13]. Similarly, amplification of the MYC oncogene has been reported in BLCA [14, 15]. The c-Myc oncoprotein is a “hypertranscription factor” that regulates transcription of at least 15% of the entire genome and controls various tumor phenotypes, including tumor cell proliferation, invasion, cell survival, genomic instability, angiogenesis, metabolism and immune evasion [13]. c-Myc is a major regulator of aerobic glycolysis, as it binds directly to a classical E-box sequence to transcribe almost all glycolytic genes [34].

The role of glycolysis in tumor progression and the central role of c-Myc in glycolysis and cancer have led to an increased interest in selective targeted therapy for tumor glucose metabolism disorders and c-Myc deregulation. One such therapy is 2-deoxy-D-glucose (2DG), a glucose analog that competitively inhibits glucose uptake and accumulates intracellularly. It then noncompetitively inhibits hexokinase (HK) and competitively inhibits phosphoglucose-isomerase (PGI). As 2DG targets glucose metabolism in tumor cells, it leads to insufficient energy supply and shows significant antitumor effects [35]. However, its clinical efficacy is diminished due to the large amount of natural glucose present in the circulation. While targeted therapies for other metabolic enzymes of tumor glycolysis are still in their early stages, few molecular targets can enter clinical trials to achieve the desired efficacy [36]. Regarding c-Myc, numerous animal experiments have demonstrated that c-Myc inactivation can cause tumor regression [37, 38]. Nonetheless, inhibiting c-Myc with small molecules is challenging since it lacks a specific active site similar to kinases. In addition, c-Myc is a transcription factor that localizes and functions in the nucleus, making it challenging to target with antibodies. Furthermore, since c-Myc is essential for normal growth and development, non-tumor-selective inhibition may result in severe toxicity to normal tissues. Thus, indirect approaches to inhibit c-Myc, such as targeting MYC transcription, translation, stability, the MYC/MAX complex, and synthetic lethality with MYC have been examined [39, 40].

**Fig. 8:**
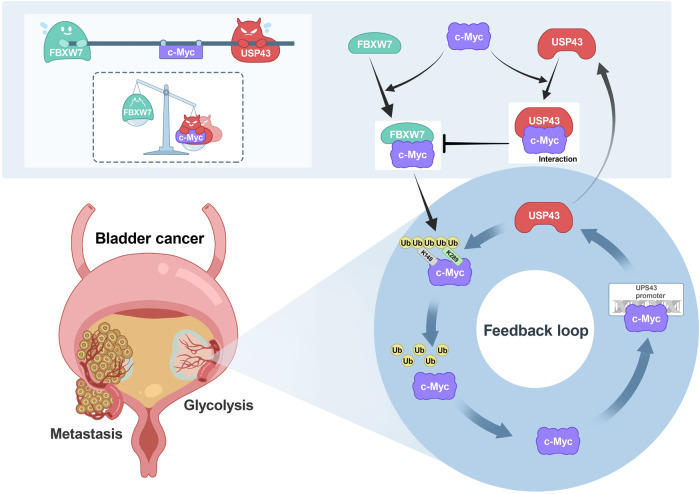
A simplified schematic diagram showing that the USP43/c-Myc positive feedback loop promoted glycolysis and metastasis in bladder cancer. USP43 stabilizes c-Myc by deubiquitinating c-Myc at K148 and K289 primarily through deubiquitinase activity. Moreover, upregulation of USP43 protein in BLCA increased the chance of interaction with c-Myc and interfered with FBXW7 access and degradation of c-Myc. In turn, stable c-Myc can function as a transcription factor that activates USP43 transcription. The dysregulation of the USP43/c-Myc positive feedback loop leads to abnormal glycolysis and the accumulation of c-Myc protein, both of which contribute to the malignant behaviors of BLCA.

The stability of the c-Myc protein is tightly regulated by the ubiquitin‒proteasome system [41]. In our current study, we screened a siRNA library targeting deubiquitinating enzymes (DUBs) to identify DUBs that positively regulate glycolysis and c-Myc transcriptional activity. We found that USP43 is a crucial deubiquitinase that controls both glycolysis and c-Myc transcriptional activity in BLCA. In particular, USP43 removes the polyubiquitination chains of c-Myc, preventing its degradation through the ubiquitin-proteasome pathway. Stable c-Myc can function as a transcription factor to activate USP43 transcription. This partially explains the upregulation of USP43 in BLCA tissues, wherein amplified c-Myc increases *USP43* mRNA levels. This study revealed a positive feedback loop between USP43 and c-Myc in BLCA. The dysregulation of this loop results in aberrant glycolysis and the accumulation of c-Myc protein, both of which contribute to the malignant behaviors of BLCA. These results suggest that USP43 is critically involved in regulating glycolysis and c-Myc activity in BLCA and therefore holds promise as a potential therapeutic target for disrupting the USP43/c-Myc circuit and regulating BLCA behavior (Fig. 8).

Several deubiquitinases for c-Myc have previously been identified, including USP28 and USP38, which function through FBXW7 to interact with c-Myc, protecting it from degradation [29, 30]. Other deubiquitinases, such as USP37, USP36, USP13, USP22, USP16, USP29, and OTUB1, have also been reported to deubiquitinate c-Myc [22, 23, 42–46]. As a result of this study, we identified USP43 as a c-Myc deubiquitinase. We found that USP43 directly cleaves the polyubiquitin chains of c-Myc, protecting it from degradation. Additionally, the interaction between USP43 and c-Myc reduces the proximity of FBXW7 to c-Myc, indirectly stabilizing it. Although previous reports have suggested that USP43 functions through its deubiquitinase activity [47–49], we also found that loss of this activity can still stabilize c-Myc. These findings suggest that the two functions of USP43 operate in coordination to maintain c-Myc as an oncoprotein and promote tumor progression (Fig. 8). Similarly, EZH2, which is known to play an oncogenic role through its methyltransferase activity, has recently been found to stabilize N-Myc independent of this activity [50].

After conducting mutant screening, we discovered that USP43 cleaves the polyubiquitin chains of c-Myc at two specific sites, K148 and K289. Additionally, our experiments revealed that the K148R, K289R, and K148/K289R c-Myc mutants significantly decreased the ability of FBXW7 to catalyze c-Myc ubiquitination, further indicating that K148 and K289 are the main sites for c-Myc ubiquitination by FBXW7. However, the K148R, K289R, and K148/K289R c-Myc mutants only partially affected the degradation of c-Myc by SKP2, indicating that K148 and K289 are not the primary sites of c-Myc ubiquitination catalyzed by SKP2. Our results suggest that USP43 and FBXW7 play antagonistic roles in controlling the protein level of c-Myc. Specifically, they balance the ubiquitination level of c-Myc at K148 and K289, thereby determining the fate of c-Myc.

In conclusion, our study revealed the mechanisms through which USP43 regulates glycolysis and c-Myc transcriptional activity. The findings of this study indicated that USP43 is a potential target to develop targeted therapy for BLCA. However, the efficacy of drugs targeting USP43 will be dependent on their ability to inhibit the deubiquitinase activity of USP43 and degrade USP43 protein or disrupt USP43-cMyc interaction.

### Reporting summary

Further information on research design is available in the Nature Research Reporting Summary linked to this article.

### Supplementary information

Supplementary Information

Original full and uncropped Western blots

Reporting Summary

## Data Availability

The publicly available data for differential gene expression analysis and survival analysis were obtained from the online website GEPIA (http://gepia.cancer-pku.cn/index.html). The publicly available TCGA-BLCA cohort data (the data included 408 tumors, and 19 normal samples) were obtained from the GDC Data Portal website (https://portal.gdc.cancer.gov/). The publicly available target genes of c-Myc were obtained from MYC ChIP-seq data in GTRD (http://gtrd.biouml.org/#!) and hTFtarget databases (http://bioinfo.life.hust.edu.cn/hTFtarget#!/). The publicly available data showing c-Myc binding peaks in the USP43 promoter region were obtained from the Cistrome Data Browser (http://cistrome.org/db/#/). The remaining data are available within the article, Supplementary Information or Original Data file.
