# Supplementary material for: USP43 stabilizes c-Myc to promote glycolysis and metastasis in bladder cancer
Source: Cell Death Dis. 2024 Jan 13;15(1):44. doi: 10.1038/s41419-024-06446-7 (PMC10787741; doi:10.1038/s41419-024-06446-7)
Supplement: Supplementary file 1 — Supplementary Information [file 41419_2024_6446_MOESM1_ESM.pdf]

Supplementary Information

USP43 stabilizes c-Myc to promote glycolysis and metastasis in bladder cancer

Supplementary Figures: Page 2-11

Supplementary Tables: Page 12-13

Supplementary Figures S1-S9

A

Top 5 DUBs

Glucose consumption	Lactate secretion
VCPIP1	USP29
USP29	VCPIP1
UEVLD	UEVLD
USP27X	USP43
USP53	USP13

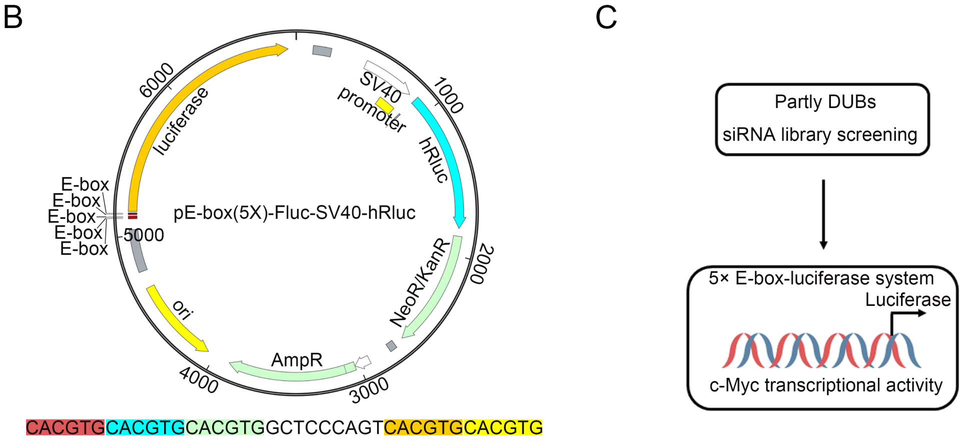

Supplementary Figure S1. Screening of DUBs that regulate glycolysis and c-Myc transcriptional activity in bladder cancer (BLCA) cells, related to Figure 1.
(A) Summary table of the top DUBs reported to regulate glycolysis. **(B)** Schematic of the constructed 5× E-box luciferase reporter reflecting c-Myc transcriptional activity. The E-box sequence (CACGTG) is the classical binding sequence of c-Myc. **(C)** Flow chart for screening DUBs that regulate c-Myc transcriptional activity.

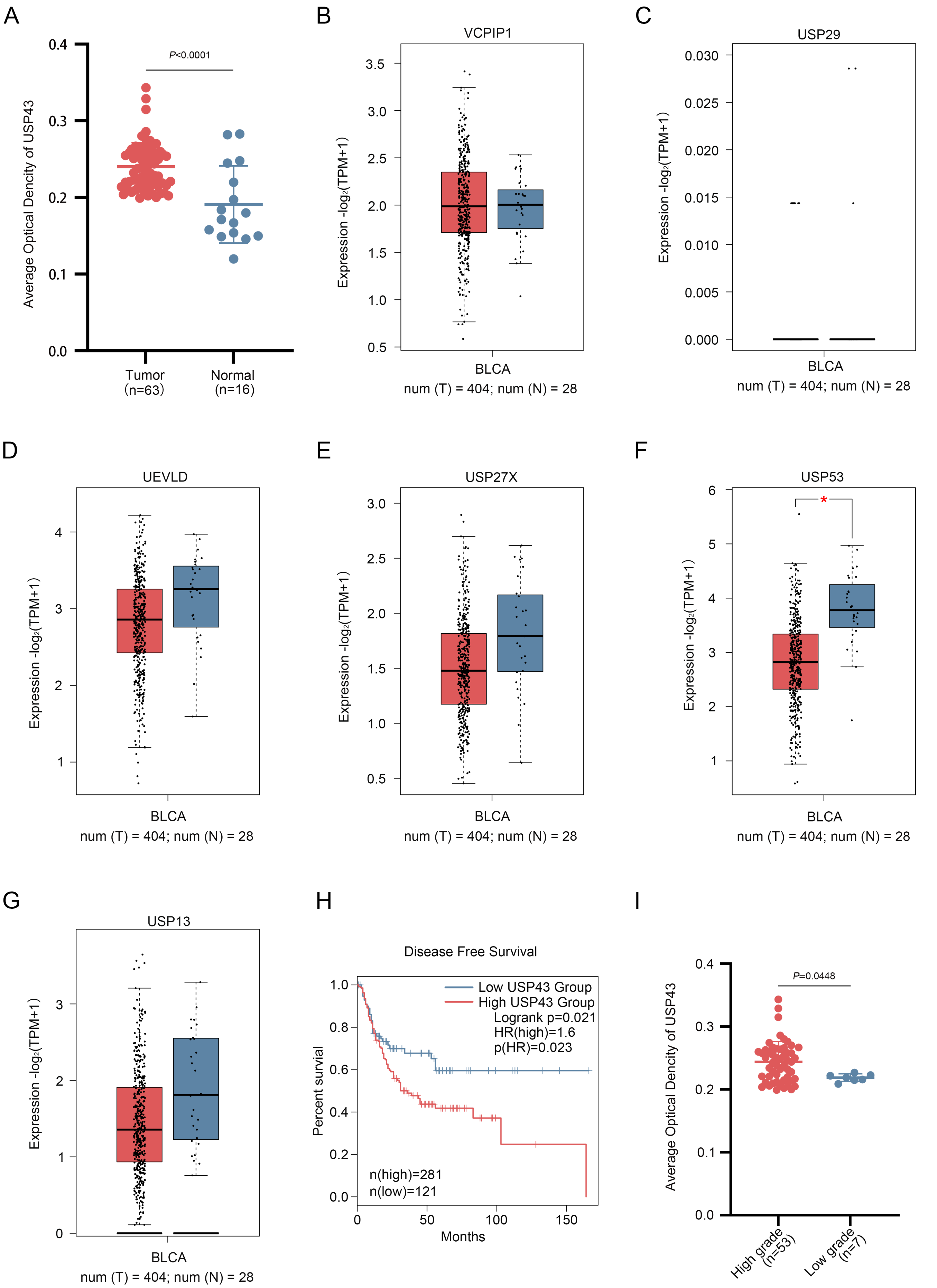
A
Average Optical Dencity of USP43
P<0.0001
0.4
0.3
0.2
0.1
0.0
Tumor
(n=63)
Normal
(n=16)
B
VCPIP1
Expression -log2(TPM+1)
BLCA
num (T) = 404; num (N) = 28
C
USP29
Expression -log2(TPM+1)
BLCA
num (T) = 404; num (N) = 28
D
UEVLD
Expression -log2(TPM+1)
BLCA
num (T) = 404; num (N) = 28
E
USP27X
Expression -log2(TPM+1)
BLCA
num (T) = 404; num (N) = 28
F
USP53
*
Expression -log2(TPM+1)
BLCA
num (T) = 404; num (N) = 28
G
USP13
Expression -log2(TPM+1)
BLCA
num (T) = 404; num (N) = 28
H
Disease Free Survival
Low USP43 Group
High USP43 Group
Logrank p=0.021
HR(high)=1.6
p(HR)=0.023
Percent survial
n(high)=281
n(low)=121
Months
I
Average Optical Dencity of USP43
P=0.0448
High grade
(n=53)
Low grade
(n=7)

Supplementary Figure S2. USP43 is highly expressed in BLCA and is associated with poor prognosis, related to Figure 1.

(A) Immunohistochemical analysis of the BLCA tissue microarray detected the difference in USP43 expression between tumor and normal tissues. The average optical density values were quantified by ImageJ software (tumor $n = 63$, normal $n = 16$, unpaired two-tailed Student's t-test). **(B-G)** Expression levels of several DUBs in BLCA and normal tissue at GEPIA. $|Log_2FC|$ Cutoff: 0.5, *p*-value Cutoff: 0.01. **(H)** Analysis of disease-free survival in patients with BLCA based on *USP43* expression in GEPIA. Cutoff-High (%): 30, Cutoff-Low (%): 30. **(I)** Immunohistochemical analysis of BLCA tissue microarray detected the expression of USP43 in low-grade and high-grade BLCA tissues (low grade $n = 7$, high grade $n = 53$, unpaired two-tailed Student's t-test). The *n* number represents n biologically independent experiments in each group. The data are presented as the mean ± SD (bar plots).

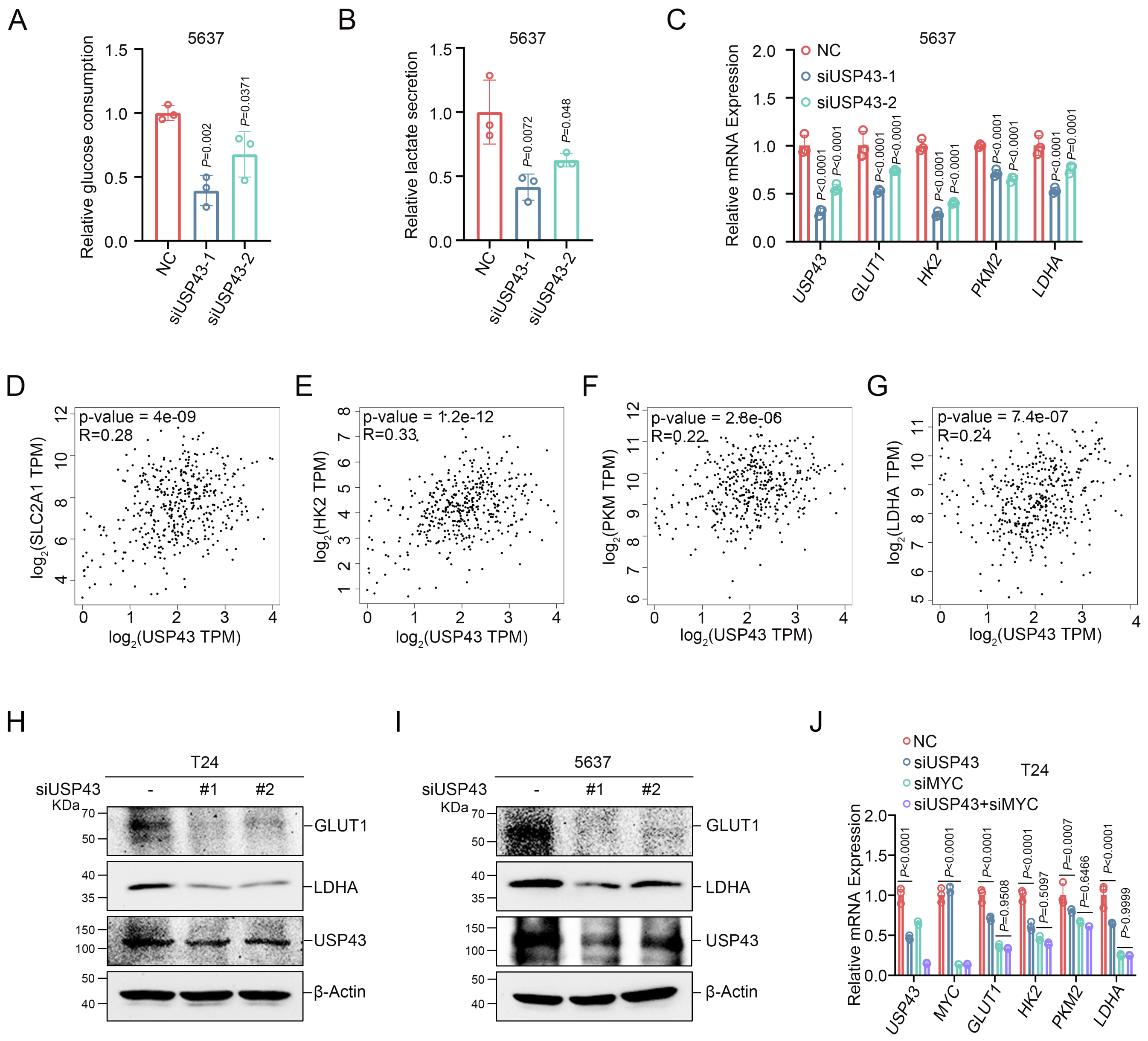

Supplementary Figure S3. USP43 regulates glycolysis in BLCA, related to Figure 1.

(A-B) The media from 5637 cells was collected for the analysis of glucose consumption (A) and lactate production (B) (A and B, *n* = 3, one-way ANOVA followed by Tukey's correction). **(C)** The mRNA levels of *USP43*, *GLUT1*, *HK2*, *PKM2*, and *LDHA* were detected using qRT-PCR after *USP43* knockdown in 5637 cells. (*n* = 3, two-way ANOVA test followed by Tukey's correction). **(D-G)** Spearman's correlation test showing a significant positive correlation between *USP43* expression and c-Myc target gene (*SLC2A1*, *HK2*, *PKM2*, *LDHA*) levels in BLCA tumor, BLCA normal and bladder tissues in GEPIA. **(H-I)** Western blot analysis was used to detect the protein levels of GLUT1 and LDHA after *USP43* knockdown in T24 (H) and 5637 (I) cells. **(J)** T24 cells were transfected with the indicated siRNA for 48 hours. The mRNA levels of *USP43*, *MYC*, *GLUT1*, *HK2*, *PKM2*, and *LDHA* were detected using qRT-PCR (*n* = 3, two-way ANOVA test followed by Tukey's correction). The *n* number represents n biologically independent experiments in each group. The data are presented as the mean ± SD (bar plots).

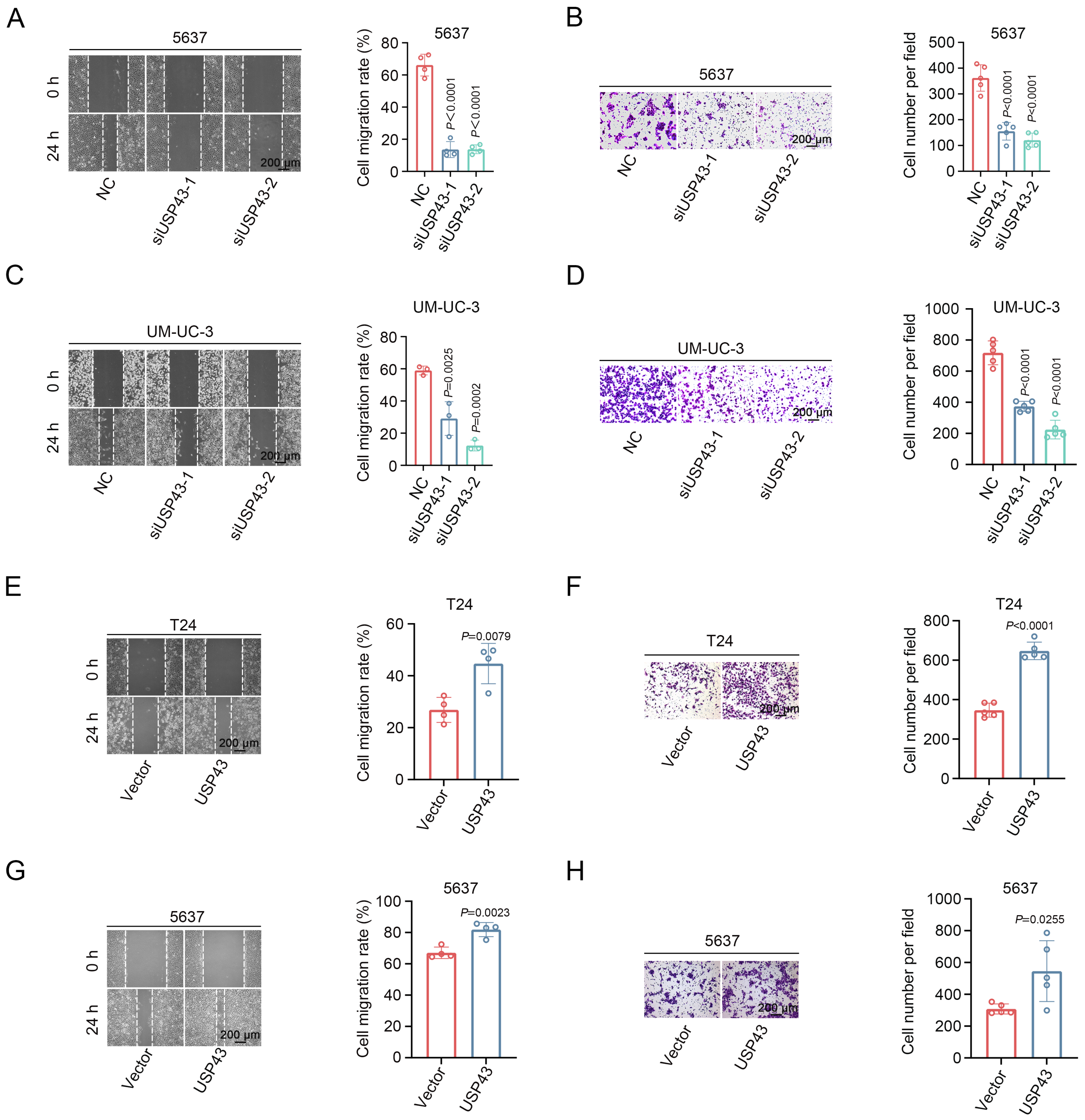

Supplementary Figure S4. USP43 regulates the migration of BLCA cells, related to Figure 2.
(A) Wound healing assay demonstrated that knockdown of *USP43* inhibited the migration ability of 5637 cells ($n = 4$). Migration rate = (wound area (0 hours) – wound area (24 hours))/wound area (0 hours). **(B)** Transwell migration assay and statistical graph after *USP43* knockdown in 5637 cells ($n = 5$). **(C-D)** Wound healing (C) ($n = 3$) and transwell migration assays (D) ($n = 5$) and their corresponding statistical plots after *USP43* knockdown in UM-UC-3 cells. **(E)** Wound healing assay and statistical graph after USP43 overexpression in T24 cells ($n = 4$). **(F)** Transwell migration assay and corresponding statistical plot after USP43 overexpression in T24 cells ($n = 5$). **(G)** Wound healing assay and statistical graph after USP43 overexpression in 5637 cells ($n = 4$). **(H)** Transwell migration assay and its corresponding statistical plot after USP43 overexpression in 5637 cells ($n = 5$). The scale bar is 200 μm. (A-D, one-way ANOVA followed by Tukey's correction; E-H, unpaired two-tailed Student's t-test). The *n* number represents n biologically independent experiments in each group. The data are presented as the mean ± SD (bar plots).

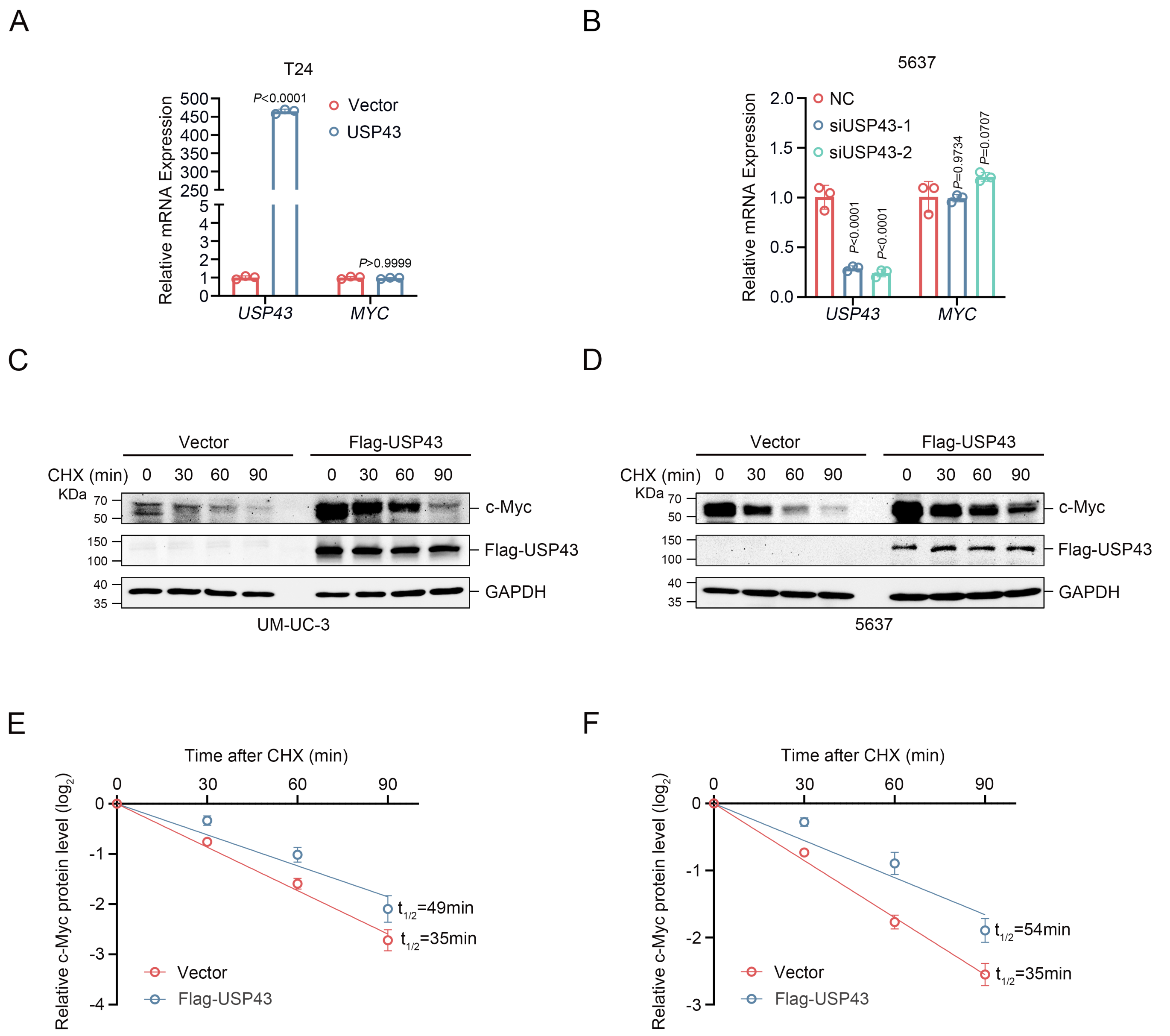

Supplementary Figure S5. Overexpression of USP43 upregulated the stability of c-Myc, related to Figure 3.

(A) *MYC* mRNA levels were measured by qRT-PCR after USP43 overexpression in T24 cells ($n = 3$, one-way ANOVA followed by Tukey's correction). **(B)** *MYC* mRNA levels were measured by qRT-PCR after *USP43* knockdown in 5637 cells ($n = 3$, one-way ANOVA followed by Tukey's correction). **(C-F)** 48 hours after USP43 overexpression, UM-UC-3 (C) and 5637 (D) cells were treated with 50 μg/mL CHX and then harvested at the indicated time points. The statistical plot (E-F) represents the intensity of c-Myc bands detected by Western blot (E and F, $n = 3$). The *n* number represents n biologically independent experiments in each group. The data are presented as the mean ± SD (bar plots).

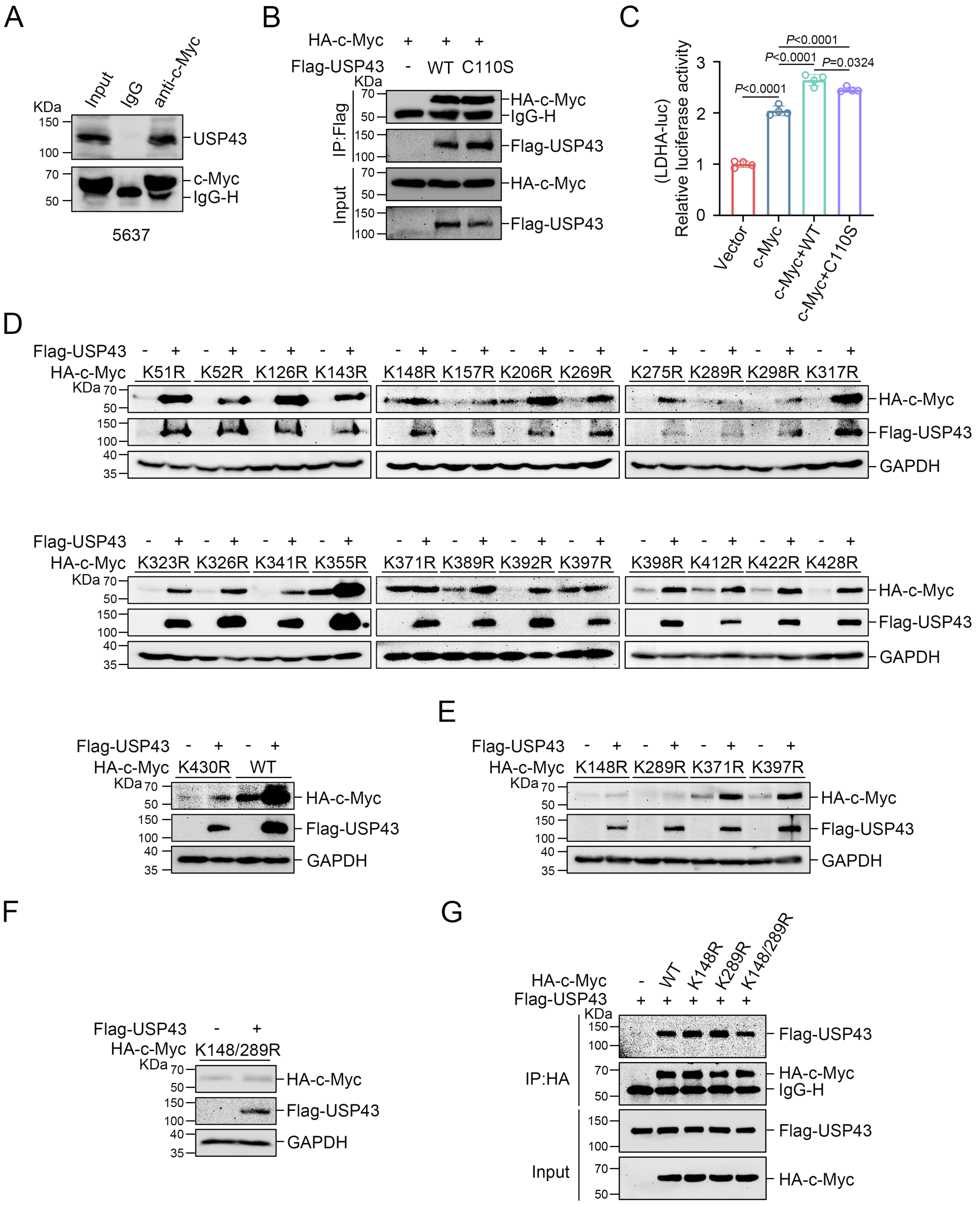

Supplementary Figure S6. USP43 targets c-Myc deubiquitination at K148 and K289, related to Figure 5.

(A) c-Myc in 5637 cell lysates was precipitated by c-Myc antibody. The interaction between endogenous USP43 and c-Myc was examined by Western blot analysis. **(B)** 293T cells were transfected according to the indicated plasmids. A co-IP assay was used to detect the interaction of wild-type USP43 and the enzyme inactivation mutant (C110S) with c-Myc. **(C)** Dual-luciferase reporter assay in 293T cells cotransfected with LDHA promoter plasmid and empty vector, c-Myc overexpression plasmid, c-Myc overexpression plasmid plus USP43 overexpression plasmid, or c-Myc overexpression plasmid plus USP43 (C110S) overexpression plasmid (n = 4, one-way ANOVA followed by Tukey's correction). **(D)** HA-tagged wild-type c-Myc and twenty-five HA-tagged c-Myc mutants were cotransfected with Flag-USP43 into 293T cells in the first round of screening, and the expression of wild-type c-Myc and c-Myc mutants was detected by anti-HA antibody. **(E)** The second round of screening focused on four c-Myc mutants, which were examined by immunoblotting after transfection as indicated in 293T cells. **(F)** Based on the results of the first two rounds of screening, a c-Myc double locus mutant was studied in the third round. **(G)** 293T cells were transfected according to the indicated plasmids. A co-IP assay was used to detect the interaction of USP43 with wild-type and various mutant c-Myc (K148R, K289R, K148/289R).

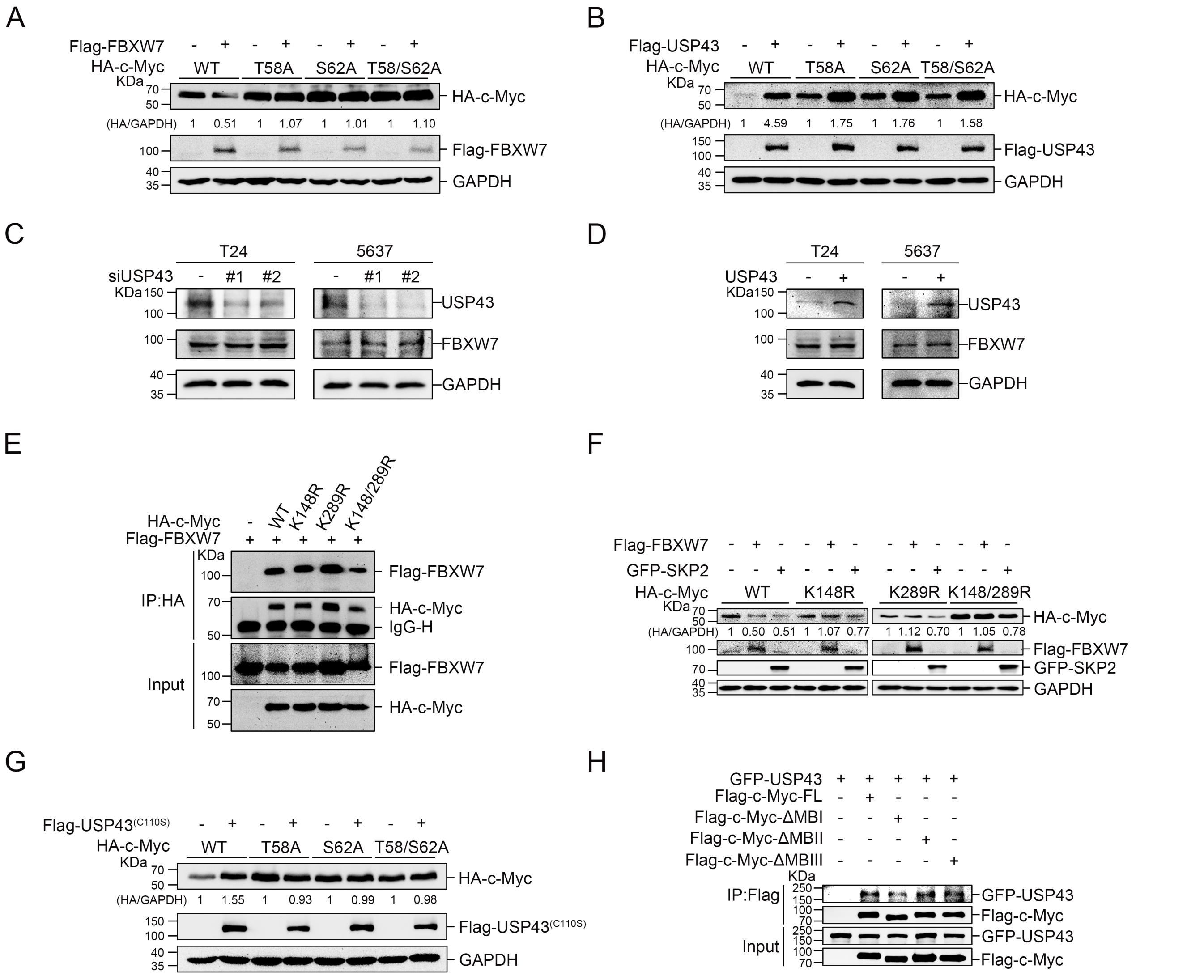

Supplementary Figure S7. USP43 antagonizes FBXW7-mediated degradation of c-Myc, related to Figure 6.

(A-B) Western blot analysis was performed after transfection as indicated in 293T cells to examine the effect of FBXW7 (A) or USP43 (B) on wild-type c-Myc and the three mutants (T58A, S62A, and T58/S62A). **(C-D)** FBXW7 was detected by immunoblot assays following *USP43* knockdown (C) and overexpression (D). **(E)** 293T cells were transfected according to the indicated plasmids. A co-IP assay was used to detect the interaction of FBXW7 with wild-type (WT) and various mutant c-Myc (K148R, K289R, K148/289R). **(F)** The effect of FBXW7 or SKP2 on wild-type c-Myc and three c-Myc mutants was analyzed by Western blotting. **(G)** Effect of USP43 enzyme inactivating mutants (C110S) on wild-type (WT) c-Myc and the three mutants (T58A, S62A, T58/S62A). **(H)** 293T cells were transfected with plasmids expressing USP43, c-Myc, or its MYC box deletion (ΔMB) mutants as shown. Anti-Flag-associated precipitates were used to detect USP43 binding.

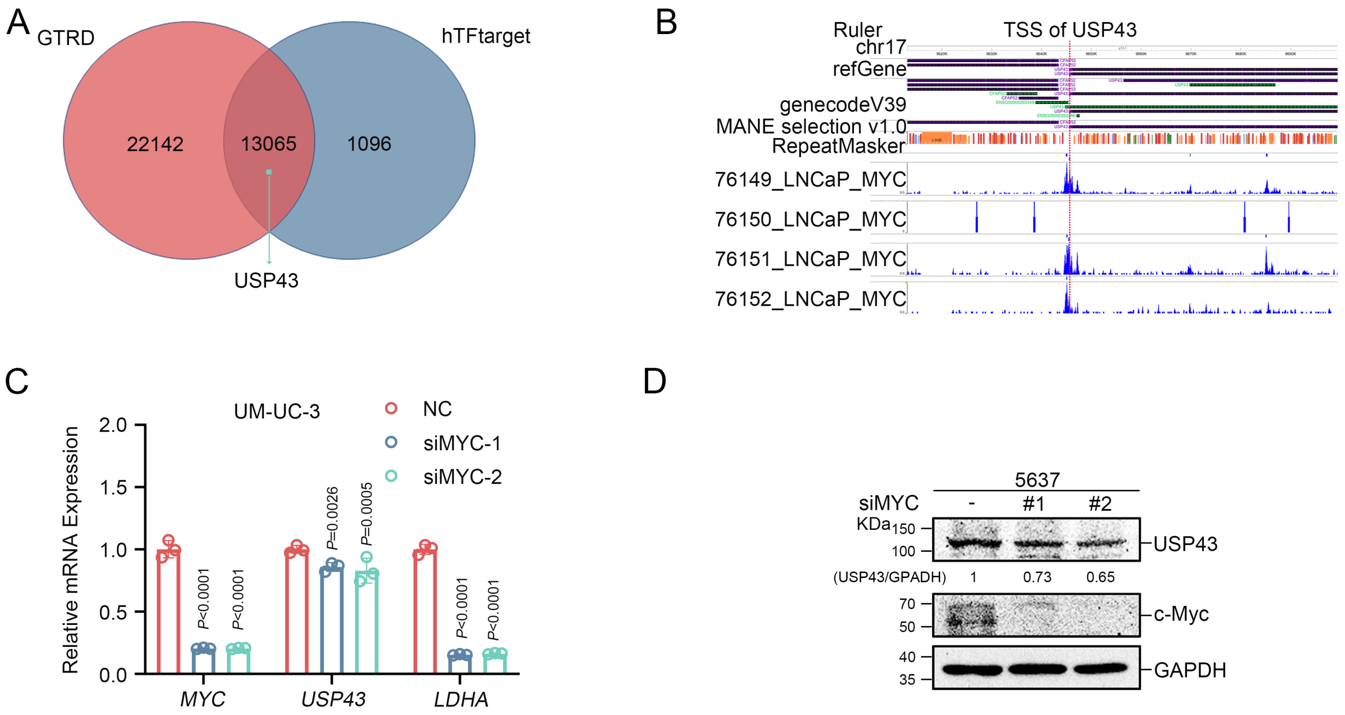

Supplementary Figure S8. c-Myc promotes the transcription of USP43, related to Figure 7.
(A) Target genes of c-Myc were obtained from ChIP-seq data in GTRD (*http://gtrd.biouml.org/#!*) and hTFtarget (*http://bioinfo.life.hust.edu.cn/hTFtarget#!/*) databases. **(B)** ChIP-seq data in the Cistrome Data Browser (*http://cistrome.org/db/#/*) showed c-Myc binding peaks on the USP43 promoter region. **(C)** UM-UC-3 cells were transfected with siRNA targeting *MYC*, and mRNA levels were detected by qRT-PCR ($n = 3$, two-way ANOVA test followed by Tukey's correction). **(D)** 5637 cells were transfected with siRNA targeting *MYC*, and protein levels were detected by Western blot analysis. The *n* number represents n biologically independent experiments in each group. The data are presented as the mean ± SD (bar plots).

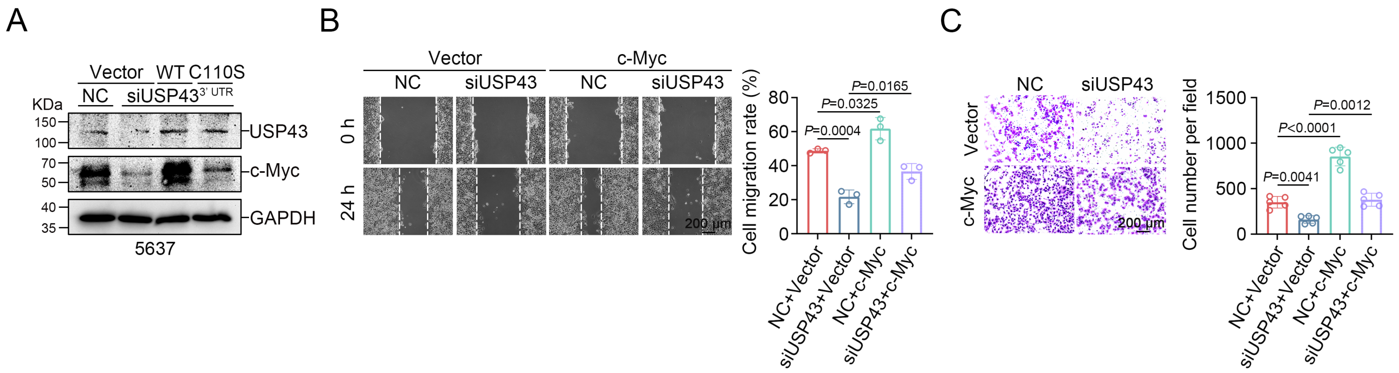

Supplementary Figure S9. USP43 regulates the migration of T24 cells by targeting c-Myc, related to Figure 7.

(A) 5637 cells were transfected with siRNA against *USP43* 3' UTR followed by reconstitution with wild-type USP43 or a deubiquitinase inactivating mutant as indicated. The protein levels were detected by Western blot analysis. **(B-C)** T24 cells were treated with siRNA targeting USP43 or c-Myc overexpression plasmid as indicated. Wound healing (B) (n = 3) and transwell migration assays (C) (n = 5) were performed to detect changes in cell migration ability (A and B, one-way ANOVA followed by Tukey's correction). The scale bar is 200 μm. The n number represents n biologically independent experiments in each group. The data are presented as the mean ± SD (bar plots).

Supplementary Tables S1-S2

Supplementary Table S1. Primer sequences for qRT-PCR and ChIP-qPCR.

Assay	Name	Forward (5' – 3')	Reverse (5' – 3')
qRT-PCR	GAPDH	GACTCATGACCACAGTCCATGC	AGAGGCAGGGATGATGTTCTG
	β-actin	GATCCACATCTGCTGGAAG	CAGCACAATGAAGATCAAGA
	USP43	CGCCTGGAAGTGTCCTCAC	CCACCTGGCAGAACCTTTTG
	MYC	GTCAAGAGGCGAACACACAAC	TTGGACGGACAGGATGTATGC
	GLUT1	CTTTGTGGCCTTCTTTGAAGT	CCACACAGTTGCTCCACAT
	HK2	GAGCCACCACTCACCCTACT	CCAGGCATTCGGCAATGTG
	PKM2	ATGTCGAAGCCCCATAGTGAA	TGGGTGGTGAATCAATGTCCA
	LDHA	ACGTGCATTCCCGATTCCTT	GGAAAAGGCTGCCATGTTGG
ChIP	P1	CCCAGCCGGGTATCTTTTTC	AGGATAAATAACATGCTTAAAAC
	P2	CACTTATTAAACAACAAGTAACAT	TCTTGCAAAATGACTTTCTCG
	P3	GGTGTAAAAAAGCAAACCAC	GAGGCAGAGTCTGTGC
	P4	CGCGCGCCTCTCACC	CATTCTGAAAGCGCCTAGTAAATGG

qRT-PCR: Quantitative reverse transcription PCR.
ChIP: Chromatin immunoprecipitation.

Supplementary Table S2. Information for the primary antibodies used in this study.

Assay	Antibody	Company	Catalog	Application
WB	GAPDH	Santa Cruz	sc-365062	1/1000
	β-Actin	Santa Cruz	sc-47778	1/1000
	USP43	Abgent	AP14283b	1/1000
	c-Myc	Abcam	ab32072	1/1000
	GLUT1	CST	12939	1/1000
	LDHA	CST	3582	1/1000
	N-Cadherin	Abcam	ab76011	1/1000
	Vimentin	CST	5741S	1/1000
	Snail	CST	3879S	1/1000
	Slug	CST	9585S	1/1000
	FBXW7	Bethyl	A301720A	1/10000
	HA-tag	Origene	TA180128	1/1000
	Flag-tag	Sigma	F1804	1/1000
	Myc-tag	ABclonal	AE010	1/1000
	GFP-tag	Santa Cruz	SC-9996	1/1000
	GST-tag	Proteintech	10000-0-AP	1/1000
	Ubiquitin	Abcam	ab7254	1/1000
	His-tag	Proteintech	66005-1-lg	1/5000
IP	c-Myc	Abcam	ab32072	1 μg/mL
	HA-tag	Origene	TA180128	1 μg/mL
	Flag-tag	Sigma	F1804	1 μg/mL
	GFP-tag	Santa Cruz	SC-9996	1 μg/mL
	IgG	Proteintech	B900610	1 μg/mL
IF	c-Myc	Abcam	ab32072	1/100
	Flag-tag	Sigma	F1804	1/100
ChIP	c-Myc	Abcam	ab32072	8 μg/10^7 cells
IHC	USP43	Abgent	AP14283b	1/100

WB: Western blot.
IP: Immunoprecipitation.
IF: Immunofluorescence.
ChIP: Chromatin immunoprecipitation.
IHC: Immunohistochemistry.
