# Supplementary material for: USP43 stabilizes c-Myc to promote glycolysis and metastasis in bladder cancer
Source: Cell Death Dis. 2024 Jan 13;15(1):44. doi: 10.1038/s41419-024-06446-7 (PMC10787741; doi:10.1038/s41419-024-06446-7)

Raw images of the immunoblotting experiments

Figure 2C

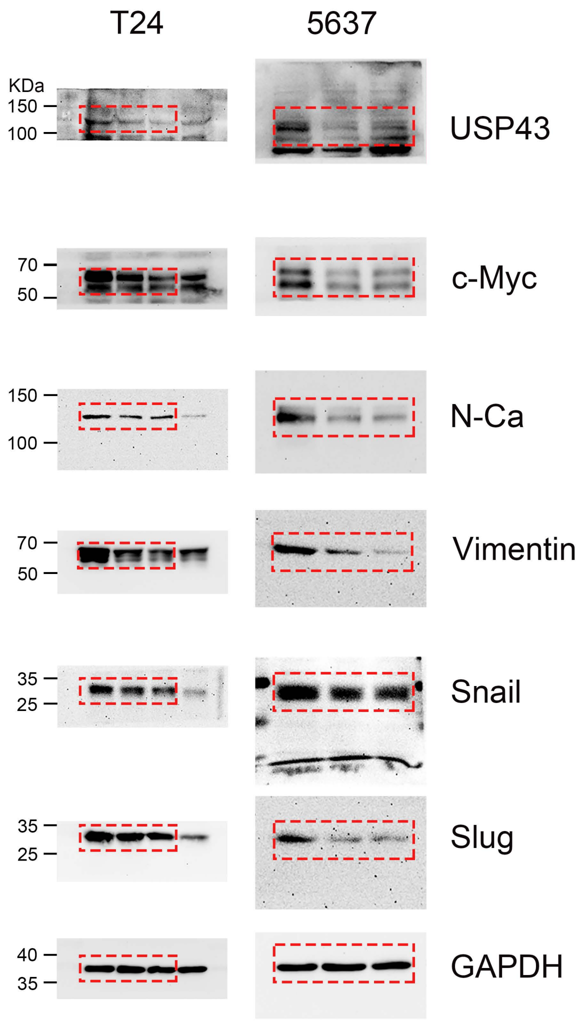

Figure 2D

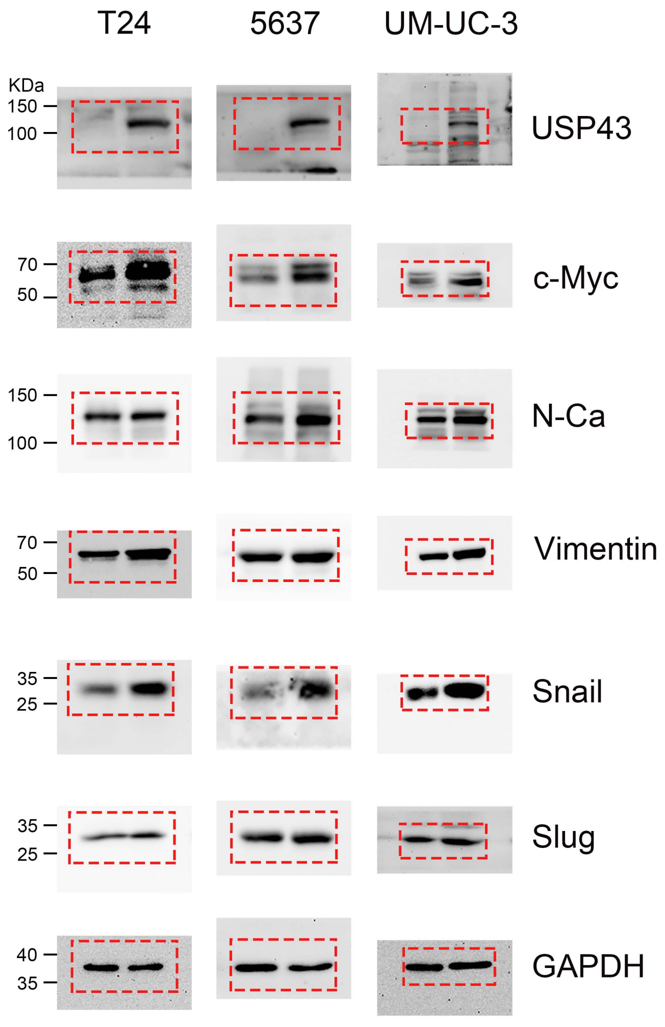

Figure 2E

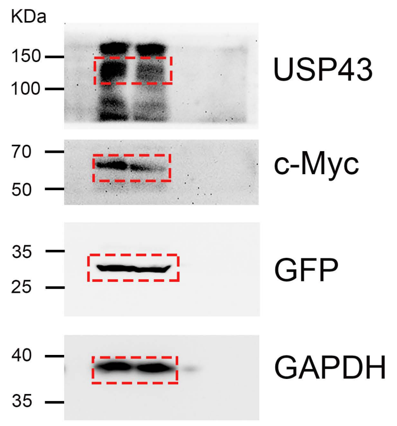

Figure 3B

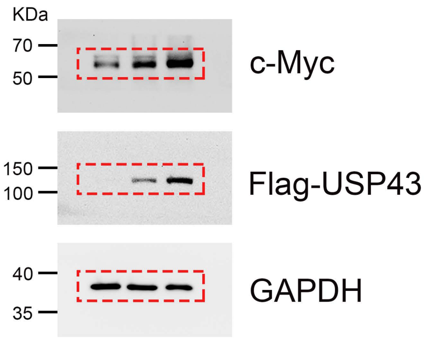

Figure 3C

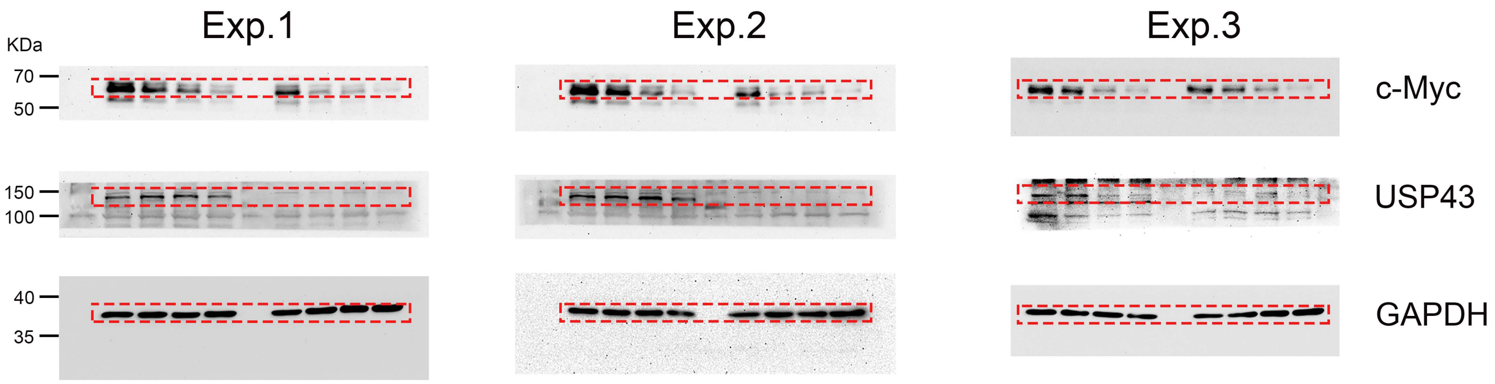

Figure 3D

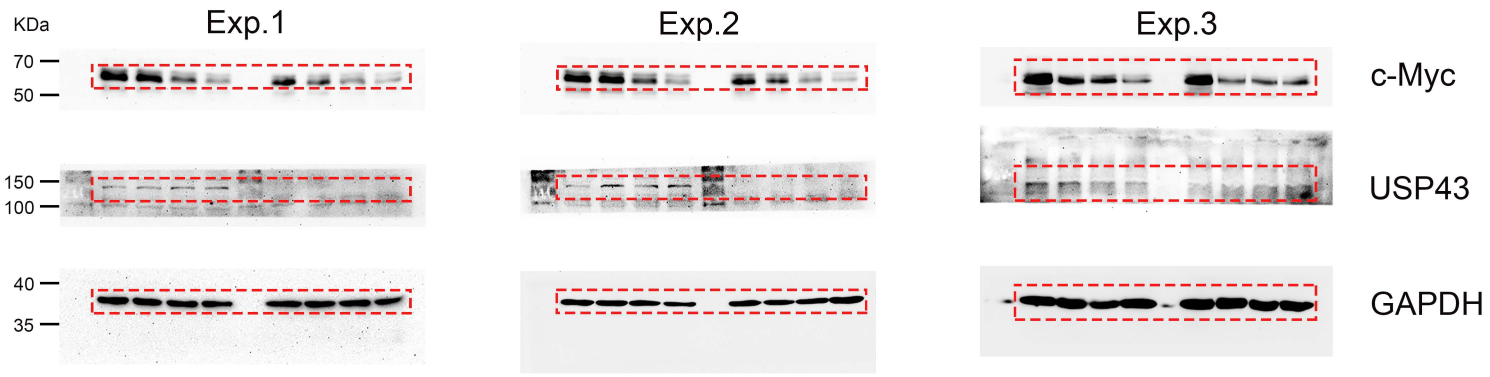

Figure 3G

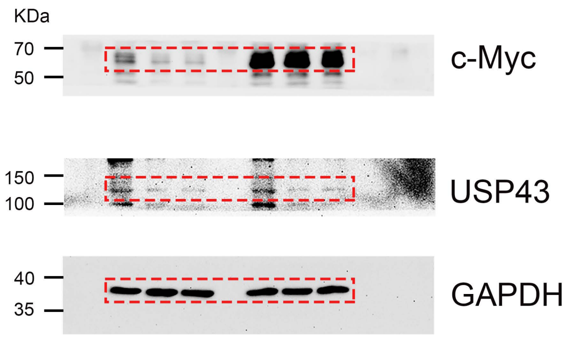

Figure 3H

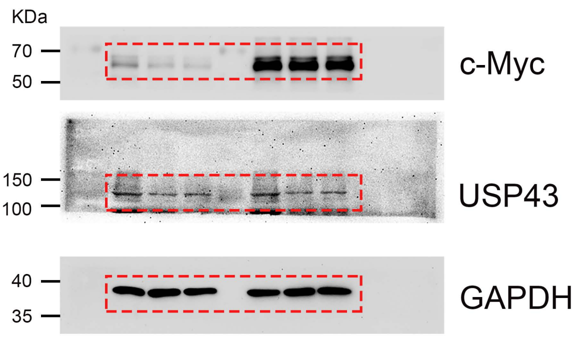

Figure 4A

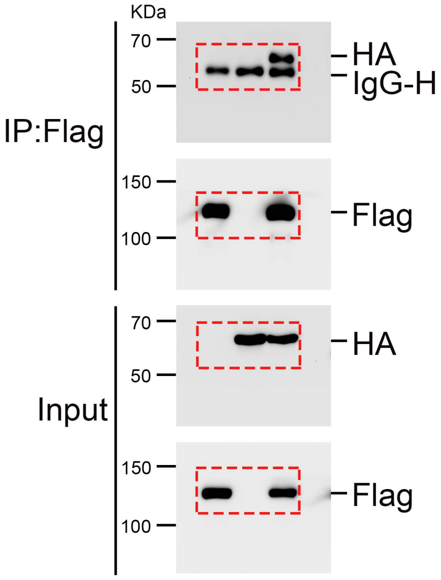

Figure 4B

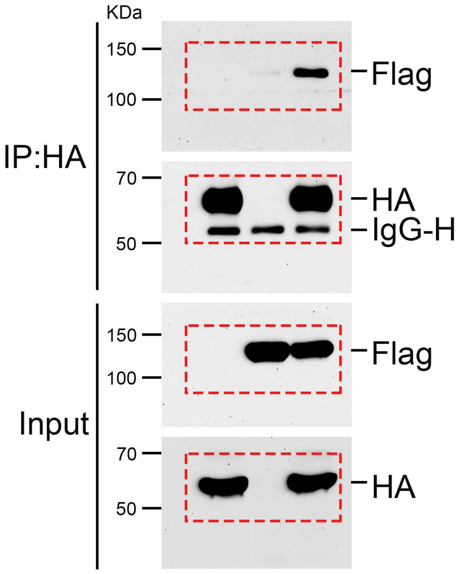

Figure 4C

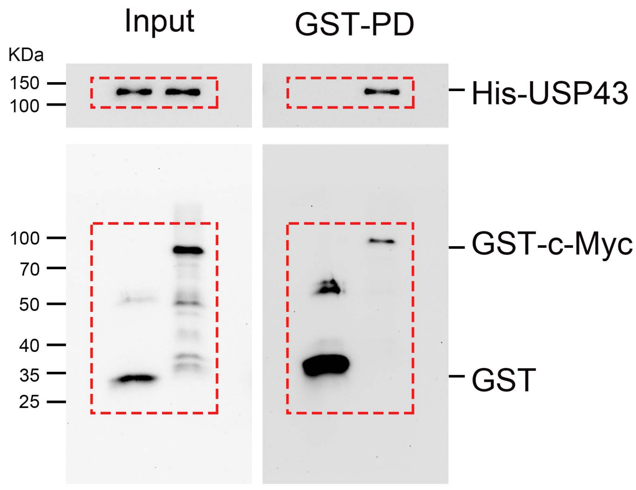

Figure 4F

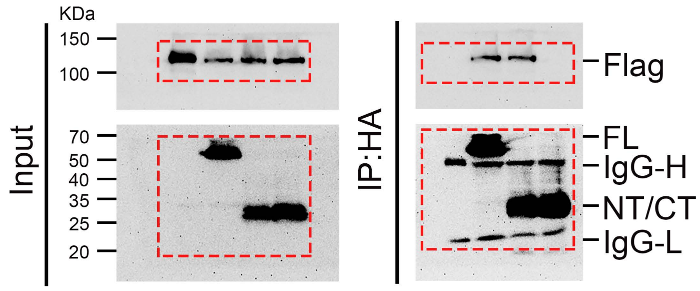

Figure 4G

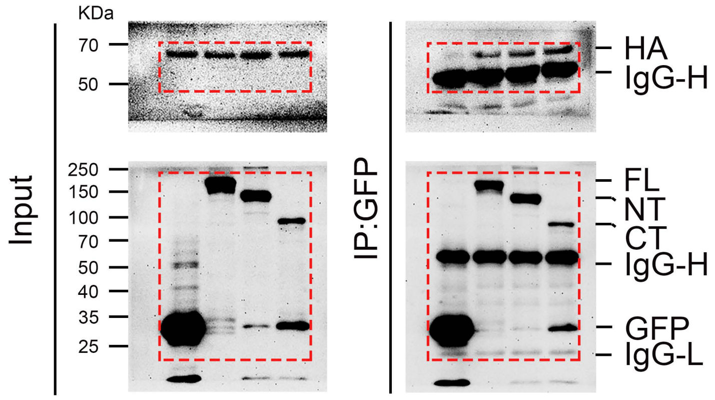

Figure 4H

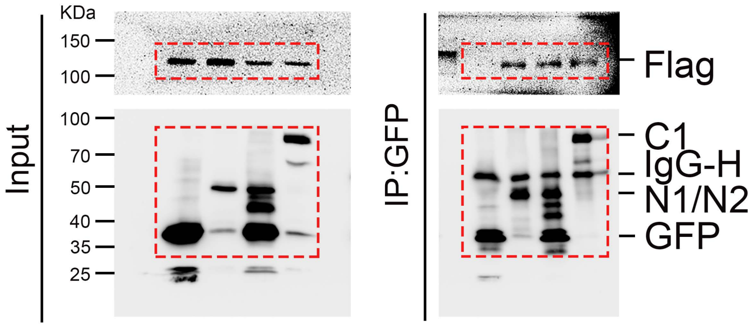

Figure 4I

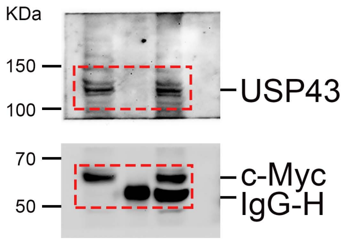

Figure 5A

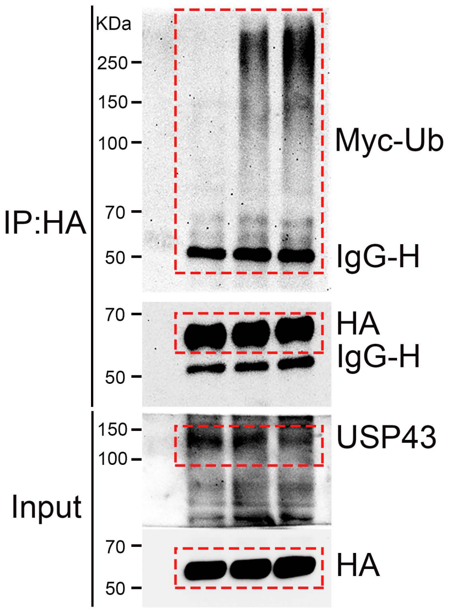

Figure 5B

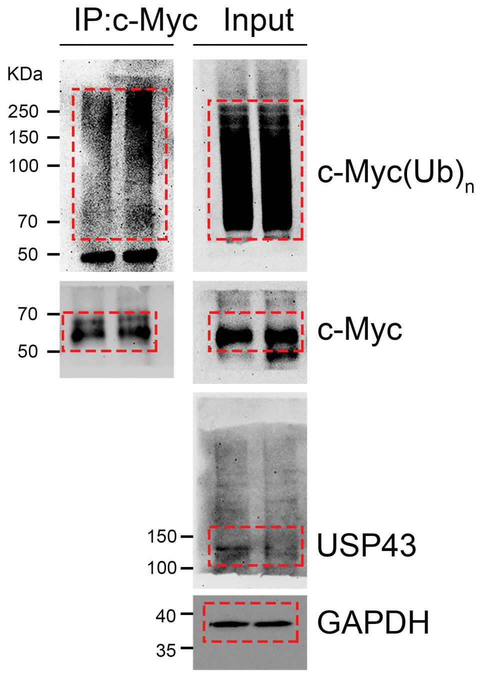

Figure 5C

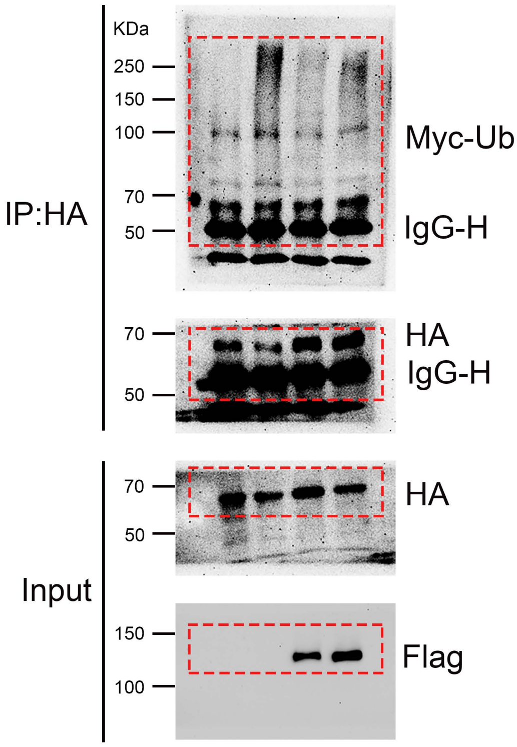

Figure 5E

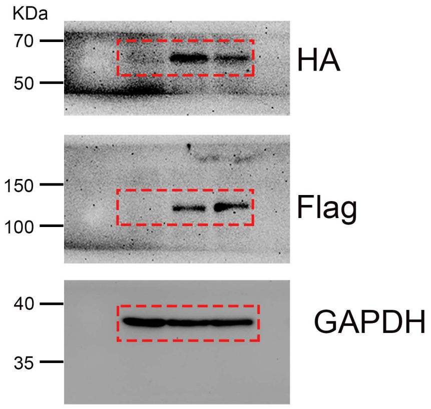

Figure 5G

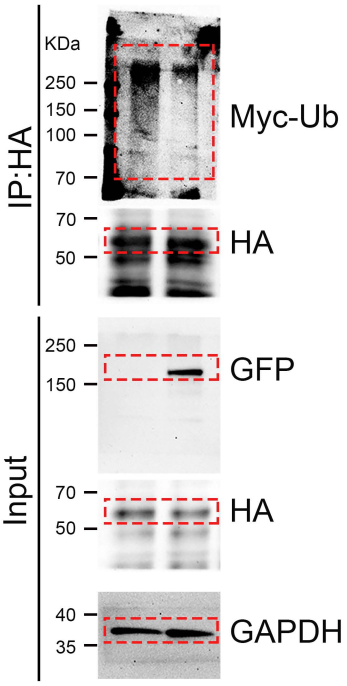

Figure 5H

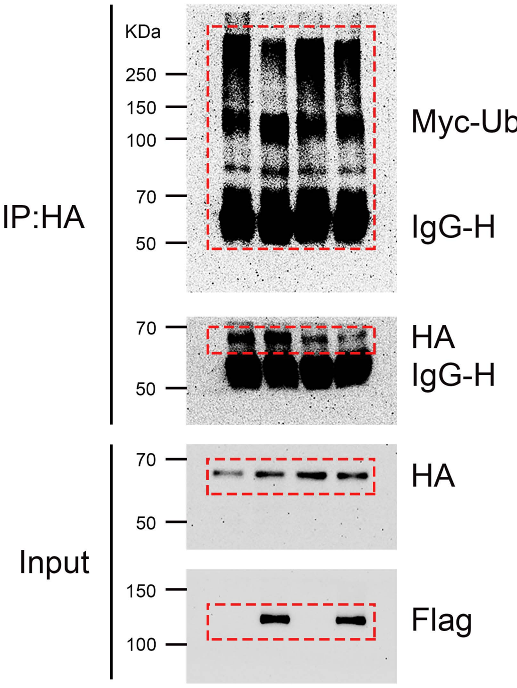

Figure 5I

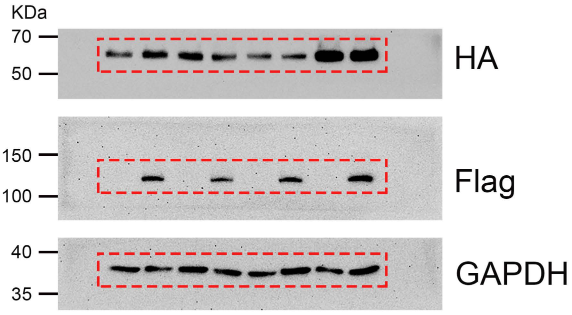

Figure 5J

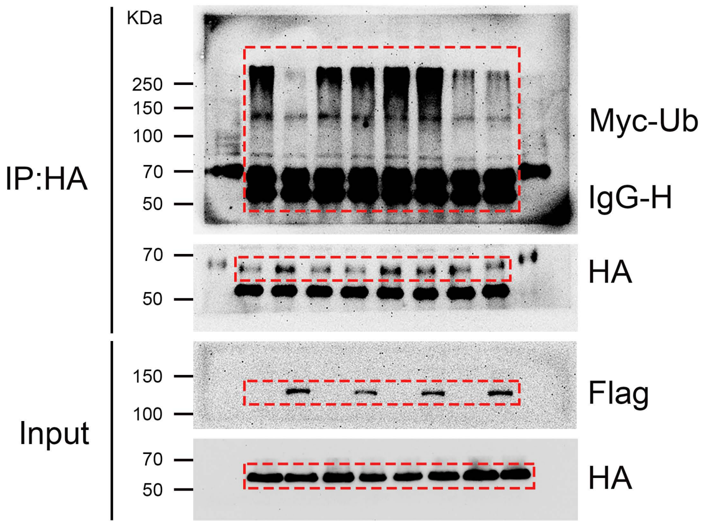

Figure 6A

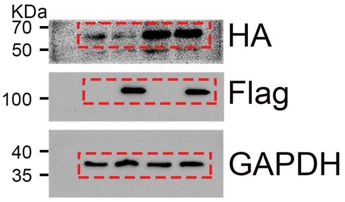

Figure 6B

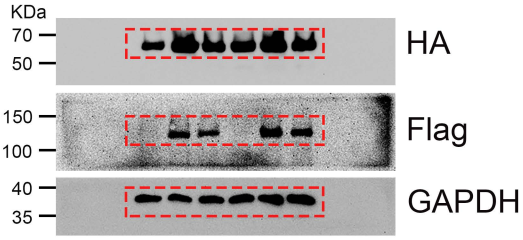

Figure 6C

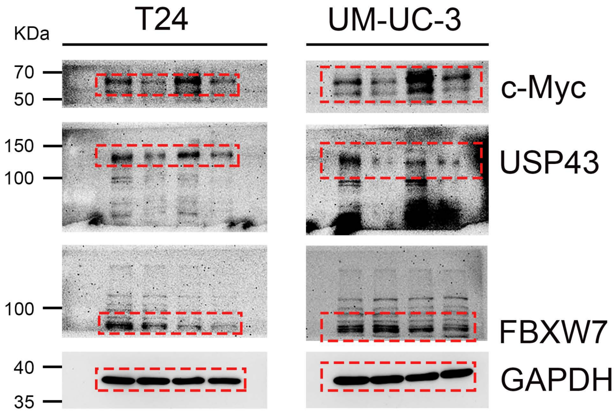

Figure 6D

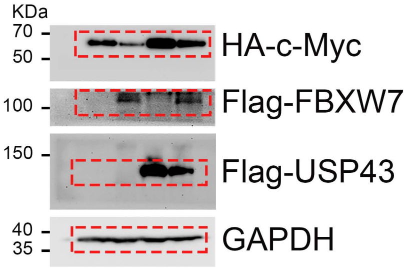

Figure 6E

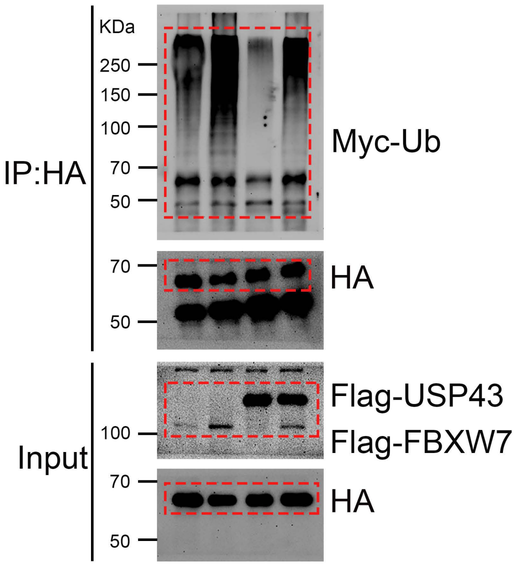

Figure 6F

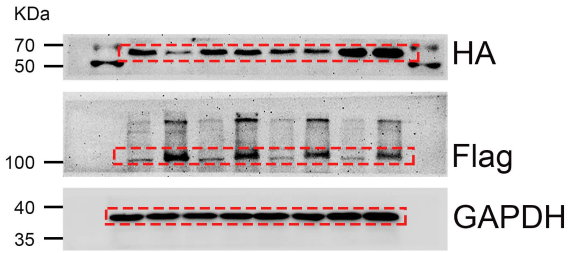

Figure 6G

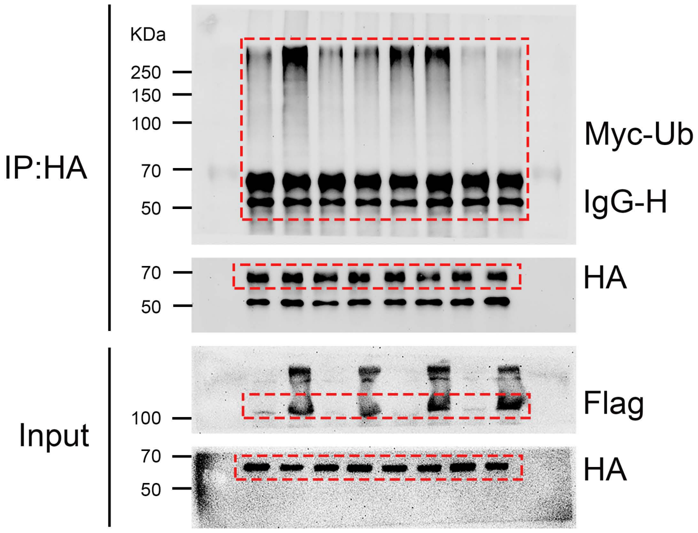

Figure 6H

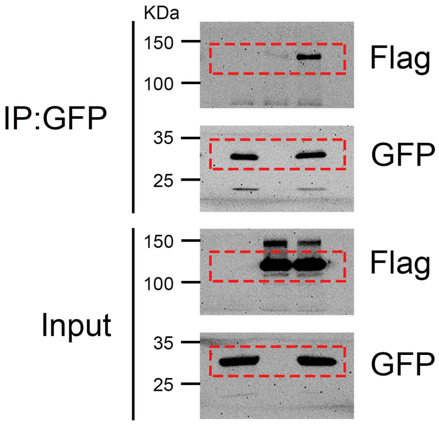

Figure 6I

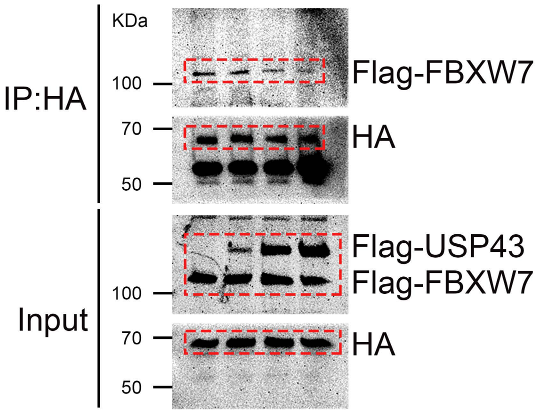

Figure S3H

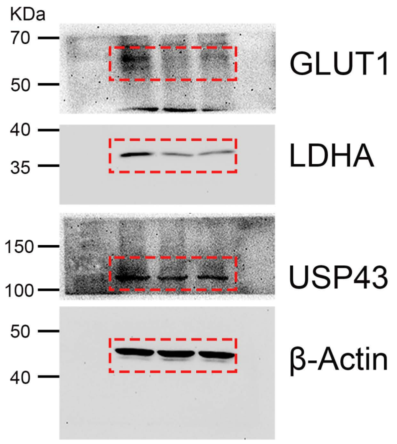

Figure S3I

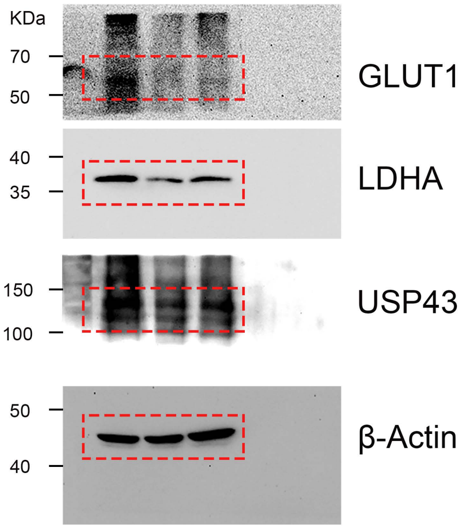

Figure S5C

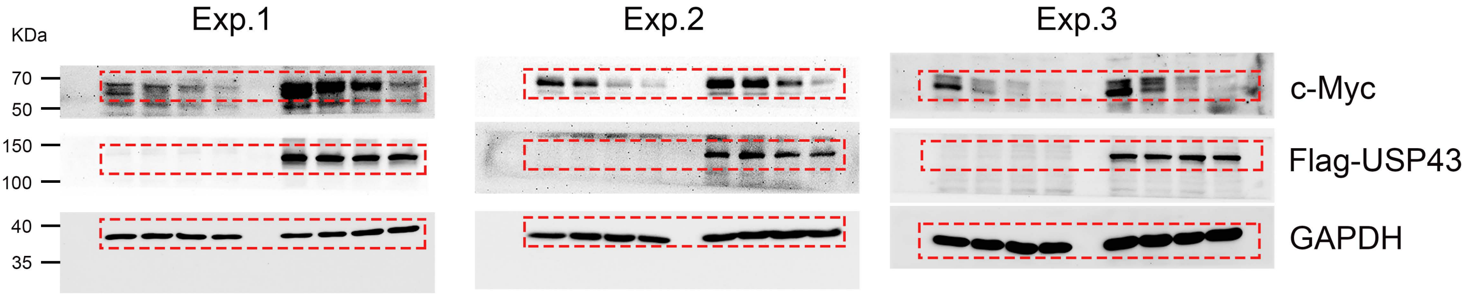

Figure S5D

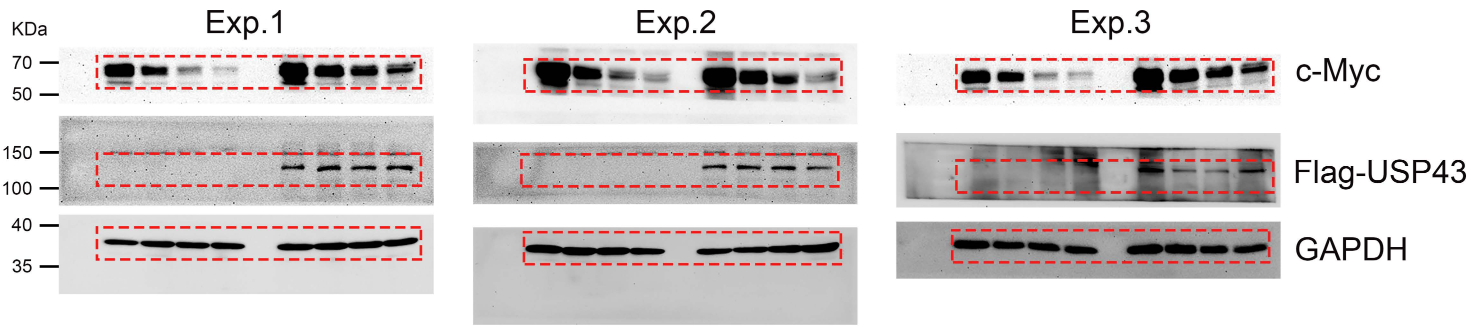

Figure S6A

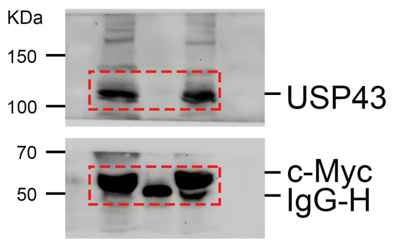

Figure S6B

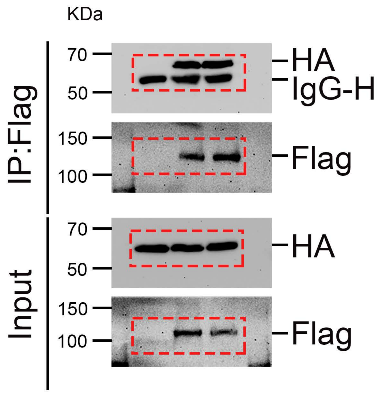

Figure S6D

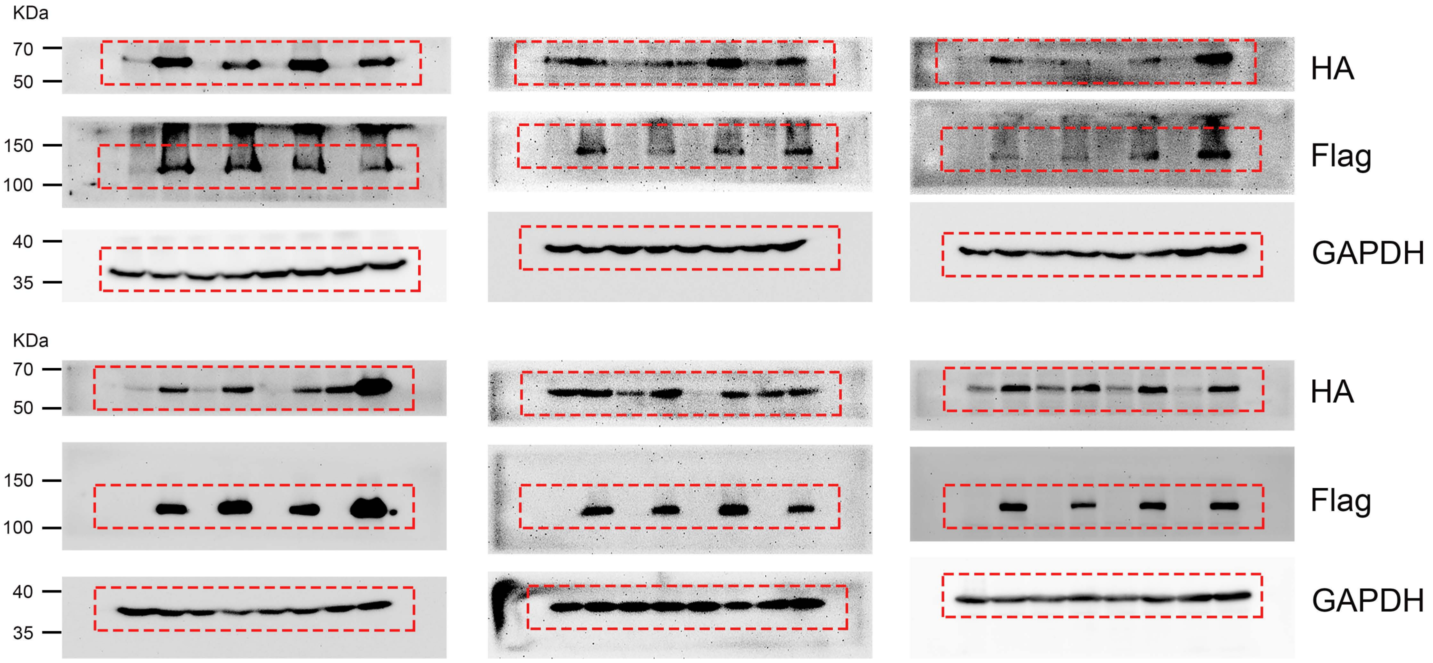

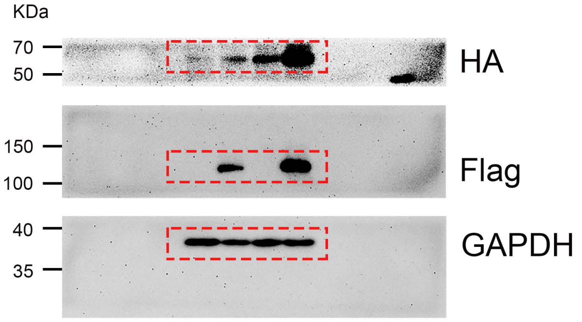

Figure S6E

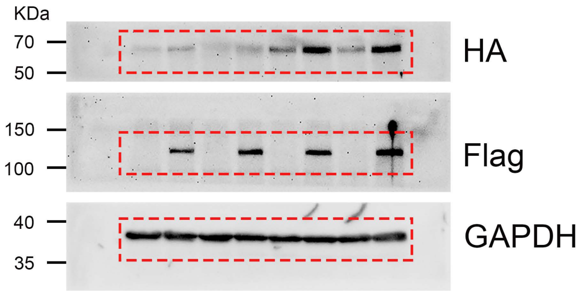

Figure S6F

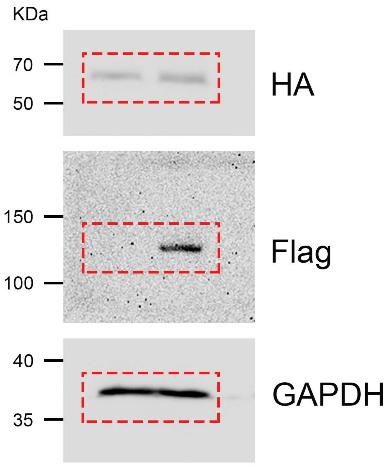

Figure S6G

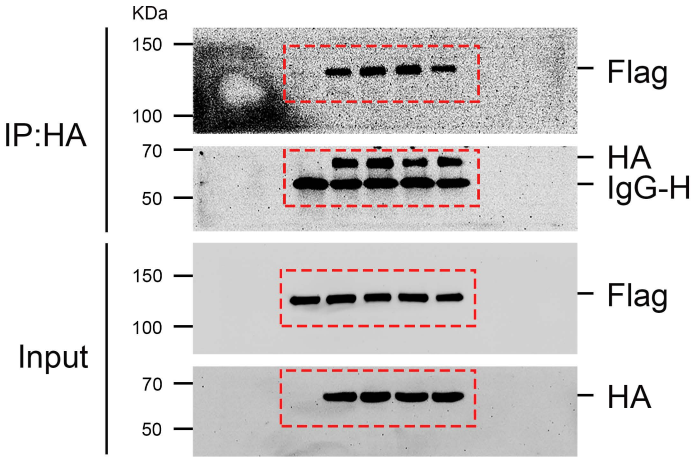

Figure S7A

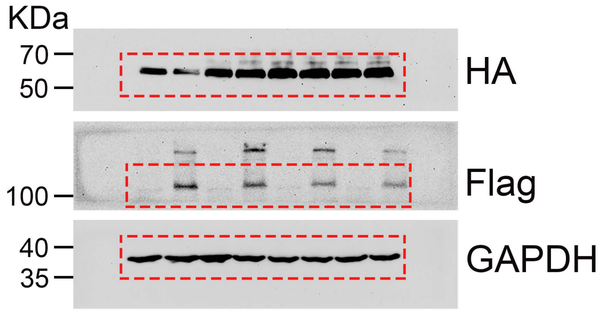

Figure S7B

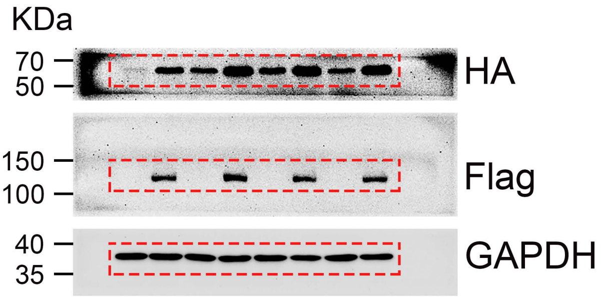

Figure S7C

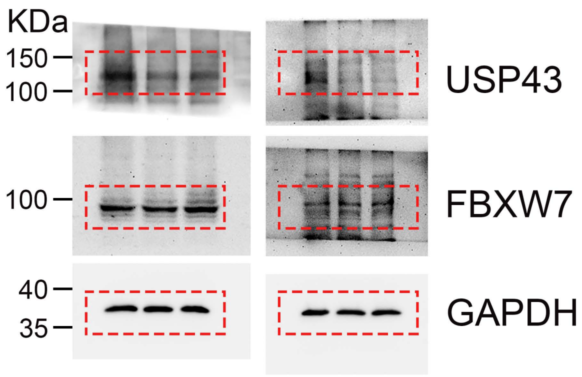

Figure S7D

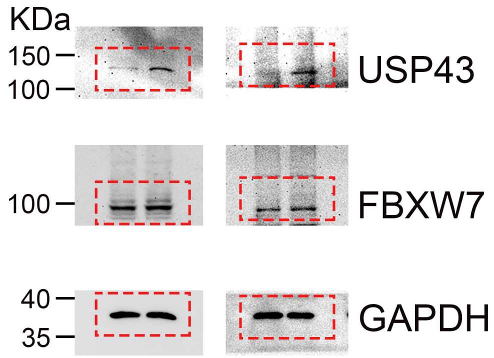

Figure S7E

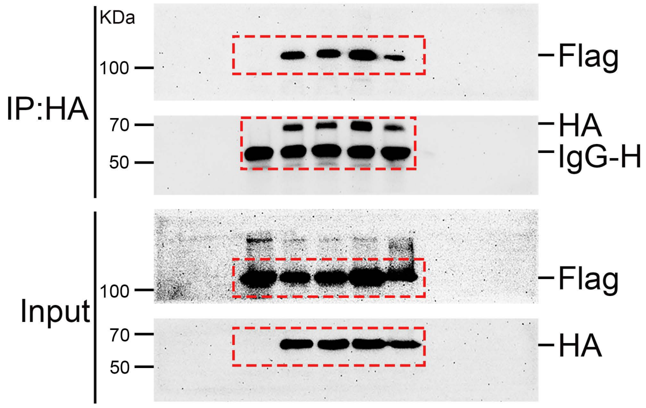

Figure S7F

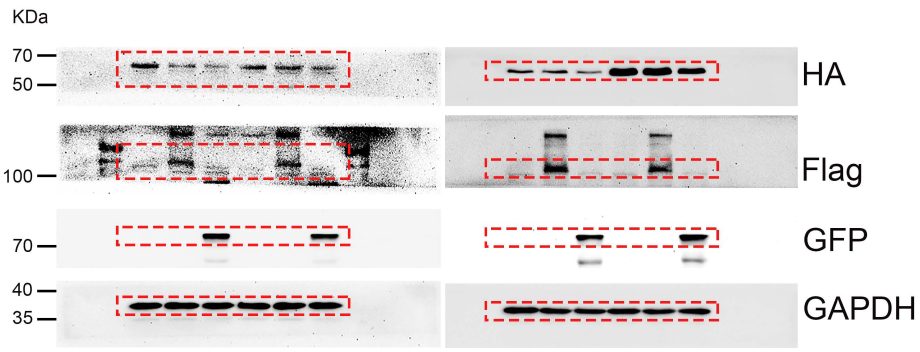

Figure S7G

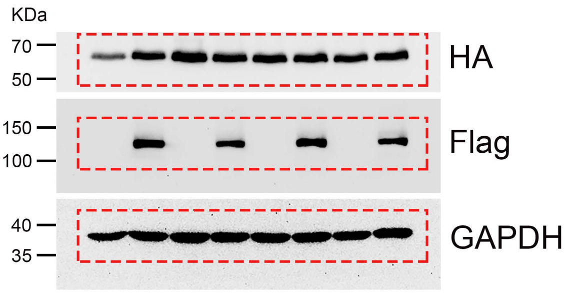

Figure S7H

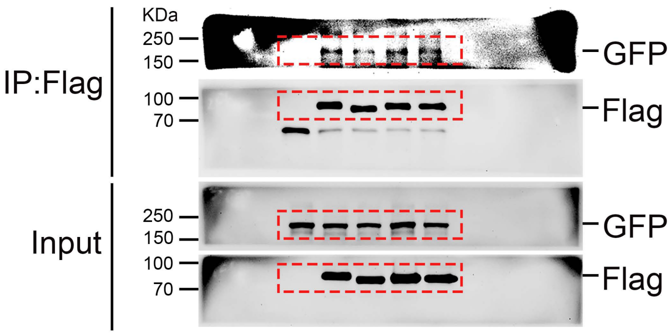

Figure S8D

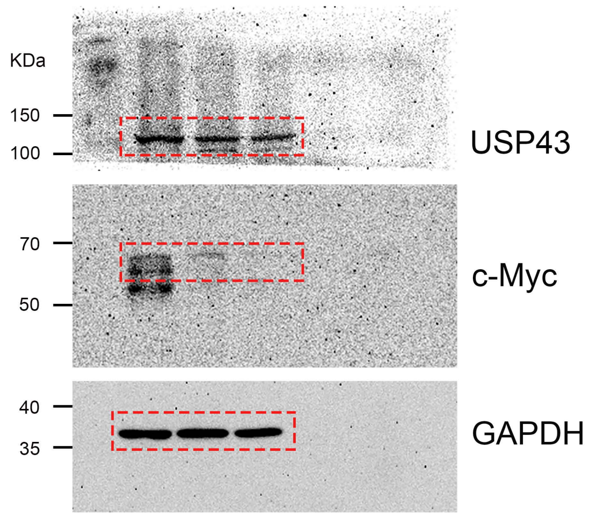

Figure S9A

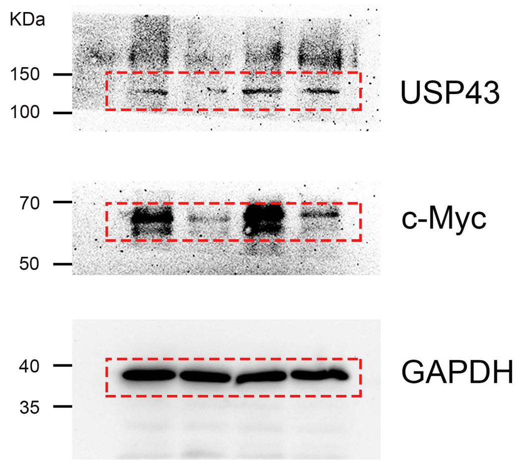

Supplement: Supplementary file 2 — Original full and uncropped Western blots [file 41419_2024_6446_MOESM2_ESM.pdf]
